# Age at death during the Covid-19 lockdown in French metropolitan regions: a non parametric quantile regression approach

**DOI:** 10.1186/s12889-024-18699-0

**Published:** 2024-05-07

**Authors:** Jonathan Roux, Marlène Faisant, Diane François, Olivier Retel, Alain Le Tertre

**Affiliations:** 1 Santé Publique France (SpF), Cellule Régionale Bretagne, Direction Des Régions 6 Place Des Colombes, Rennes Cedex, 35042 France; 2 Santé Publique France (SpF), Cellule Régionale Bourgogne-Franche-Comté, Direction Des Régions, 21035 Dijon, France

**Keywords:** Mortality, Covid-19, SARS-CoV-2, Age at death, Quantile regression, France

## Abstract

**Background:**

Lockdowns have been implemented to limit the number of hospitalisations and deaths during the first wave of 2019 coronavirus disease. These measures may have affected differently death characteristics, such age and sex. France was one of the hardest hit countries in Europe with a decreasing east–west gradient in excess mortality. This study aimed at describing the evolution of age at death quantiles during the lockdown in spring 2020 (17 March—11 May 2020) in the French metropolitan regions focusing on 3 representatives of the epidemic variations in the country: Bretagne, Ile-de-France (IDF) and Bourgogne-Franche-Comté (BFC).

**Methods:**

Data were extracted from the French public mortality database from 1 January 2011 to 31 August 2020. The age distribution of mortality observed during the lockdown period (based on each decile, plus quantiles 1, 5, 95 and 99) was compared with the expected one using Bayesian non-parametric quantile regression.

**Results:**

During the lockdown, 5457, 5917 and 22 346 deaths were reported in Bretagne, BFC and IDF, respectively. An excess mortality from + 3% in Bretagne to + 102% in IDF was observed during lockdown compared to the 3 previous years. Lockdown led to an important increase in the first quantiles of age at death, irrespective of the region, while the increase was more gradual for older age groups. It corresponded to fewer young people, mainly males, dying during the lockdown, with an increase in the age at death in the first quantile of about 7 years across regions. In females, a less significant shift in the first quantiles and a greater heterogeneity between regions were shown. A greater shift was observed in eastern region and IDF, which may also represent excess mortality among the elderly.

**Conclusions:**

This study focused on the innovative outcome of the age distribution at death. It shows the first quantiles of age at death increased differentially according to sex during the lockdown period, overall shift seems to depend on prior epidemic intensity before lockdown and complements studies on excess mortality during lockdowns.

**Supplementary Information:**

The online version contains supplementary material available at 10.1186/s12889-024-18699-0.

## Background

Severe acute respiratory syndrome coronavirus 2 (SARS-CoV-2), which led to the 2019 coronavirus disease (Covid-19), was first identified at the end of December 2019 and rapidly spread globally to become a pandemic. In spring 2020, to address this public health threat and limit its spread, several countries around the world decided to implement unprecedented mitigation measures, ranging from mobility restrictions to a global lockdown.

These measures had the desired effect of slowing the spread of the virus and preventing health systems from becoming overwhelmed. However, they also had side effects such as reductions in health care use [1], reductions in hospital admissions for all causes [2, 3] and changes in the causes of death [4]. Another major impact of this pandemic was a reduction in life expectancy in the US [5], Europe [6, 7] and several other regions of the world [8, 9]. To our knowledge, few studies have specifically examined whether the age structure of deaths has changed. A first study from the UK showed a reduction in lifespan inequality of 5 months between the two sexes during 2020, assuming a change in the age-structure of deaths [7]. A second study observed a shift in the distribution of age at death in India in 2020 compared with previous years, due to a heavy toll of deaths among adults (20–64 years) and men [9].

In France, the government decided to implement a nationwide lockdown from March 17 to May 11, 2020, mainly for the entire first wave of the pandemic (March 2 to May 31, 2020). Despite this measure, France was one of the hardest hit countries in Europe, with 29 400 deaths of patients infected with or diagnosed with COVID-19 (excluding at home deaths) during the first wave of the pandemic [10]. The use of health care services decreased from -7% to -96% compared to 2019, depending on the medical speciality, during the lockdown period [11]. This decrease was also observed for dedicated health care, such as stroke [12] or geriatrics [13]. In terms of causes of death, Covid-19 was the third leading cause of death in France in 2020 [14] and a decrease in deaths due to drowning and transport accidents was observed in France in 2020, but also in deaths due to cancer, vascular diseases, mental and nervous system diseases compared to expectations [15]. An increase in the number of deaths compared to expectations was observed at national and regional levels [10] during the first lockdown period, with an estimate of + 18% according to Santé publique France, the French national public health agency. This excess mortality was not evenly distributed across the territory [16] or across age groups [11, 17]. In fact, fewer deaths were observed in the young population and more in the older population, because of the age-dependent risk of hospitalisation and death, as shown by modelling studies [18]. Furthermore, an east–west gradient in excess mortality has been quantified in France, with eastern regions being more affected due to initial infection. For example, in Bourgogne-Franche-Comté (BFC), a region in eastern France, a higher than expected mortality was observed during the first wave with + 23% deaths (+ 1647, 95% confidence interval (95%CI) = [+ 1404; + 1882]) [19]. Conversely, in Bretagne in northwestern France, a decrease of 3% was observed compared with the expected number of deaths during the same period, namely -281 deaths, 95%CI = [-543; -26] [20]. Ile-de-France (IDF), the region with the largest population, was the hardest region hit with + 67% more deaths (+ 12 443, 95% confidence interval (95%CI) = [+ 12 047; + 12 830]) [21].

There is a lack of data on the direct impact of the mitigation period against the first wave of the Covid-19 pandemic on a change in the age distribution at death, in particular on the effect of the global lockdown. Therefore, the present study aimed to describe the evolution of the innovative outcome of age at death, based on its quantiles, during the Spring 2020 lockdown in French metropolitan regions. In the present article, we focus on three representatives of the epidemic variations in the country: Bretagne (low impacted region), BFC (high impacted region) and IDF (most impacted region) (Results for the 9 other metropolitan regions are presented in Additional file 1).

## Methods

### Mortality data

Data were extracted from the publicly available death database of the French National Institute of Statistics and Economic Studies (Insee) from January 1st, 2011 to August 31st, 2020 [22]. These data provide up-to-date information on all deaths by age, sex, date of death and department of death, *i.e.* not necessarily the place of residence at death, occurring in France. For this study, we extracted all deaths that occurred in French metropolitan regions.

### Statistical analysis

We compared the age distribution of mortality observed during lockdown period with the expected one, that should have been observed in the absence of lockdown, during this period. The aim was to determine whether, during this specific period, the age distribution of deaths was affected compared with the usual distribution.

We used a Bayesian non-parametric quantile regression [23] to assess change in age at death distribution as a function of time and lockdown. Quantile generalized additive models (QGAMs) aim at modelling the effect of the predictors on a specific quantile of the dependent variable, rather than on the more common choice of the mean value of the dependent variable. In mathematical terms, a QGAM model can be expressed in the following way:

$${Q}_{t}\left(Y\right)={f}_{1}({x}_{1})+{f}_{2}({x}_{2}) + ... +{f}_{i}({x}_{i})$$

where ${Q}_{t}(Y)$ is a quantile of choice of the dependent variable, here a quantile of age, and ${f}_{i}({x}_{i})$ is a smooth function of an independent variable, time for example. The main advantage of quantile regression is its robustness for outliers. Furthermore, this model allows the relationship between the predictors and the values of dependent variable to be explored across the distribution, which is what this research aims to do. We chose a wide range of 13 quantiles from the 1st to the 99th percentile (each decile, plus quantiles 1, 5, 95 and 99). The latter helped us to determine whether the effect of the predictor of interest, *i.e.* lockdown, had a protective or adverse impact from the youngest to elderliest people, although we cannot exclude a confounding impact of the epidemic itself without being able to make a clear distinction between the two.

Each quantile was modelled as a function of its trend, both long term and seasonal. The modelling of this trend was based on a thin plate penalised spline function of time with the smoothing parameter estimated automatically by marginal loss minimisation. Lockdown period was specifically modelled with a thin plate spline function and with sufficient flexibility to capture variations during this period. Thus, the parameter of interest, the trend including seasonality, was not affected by this specific period. Our model can be summarised as follows:

$$Q_t\left(Age\right)=s(time,by={lockdown}_{FALSE})+s(time,by={lockdown}_{TRUE})-1$$

with time being calendar date. By not including the main effect for lockdown and the intercept (- 1 in the equation), the smooth function of time by lockdown levels incorporates these effects and could then be interpreted directly. The model thus provided the expected quantile during lockdown period with a 95% credibility interval, incorporating the uncertainty associated with the selection of smoothing parameters. As the age of death distribution differs by sex, we also stratified the analysis by sex to allow specific estimates separately.

We expressed the results as age difference for each quantile during lockdown period compared to expected ones in the absence of lockdown or pandemic. We also estimated the shift in quantile distribution at age constant. To help interpret a change in quantiles, we represented the crude death pyramid comparing the number of deaths during lockdown and the average number of deaths over the same period in the previous 3 years. All analyses were achieved using R software (version 4.2.3) [24] and QGAM package [25].

## Results

Over the study period (January 2011-August 2020), the number of deaths ranged from 244 097 in Centre-Val de Loire (CVL) to 719 086 in IDF. In Bretagne, 328 240 deaths were recorded and 280 626 in BFC. During the lockdown period (17 March – 11 May, 2020), it varied between 4654 in CVL and 22 346 in IDF, with 5457 and 5917 deaths in Bretagne and BFC, respectively. The average age at death during the lockdown was higher in all regions compared to the whole study period (79.9 *vs.* 78.5 in Bretagne, 81.4 *vs.* 79.0 in BFC and 79.7 *vs.* 76.2 in IDF, respectively). Compared to the three previous years (2017–2019) in the same period, an excess mortality of + 2.7% was observed in Bretagne (minimum across all regions), + 32.7% in BFC and + 102.0% in IDF (maximum across all regions) during lockdown. The results for all the 12 metropolitan French regions are presented in Supplementary Table 1, Additional File 1.

**Fig. 1:**
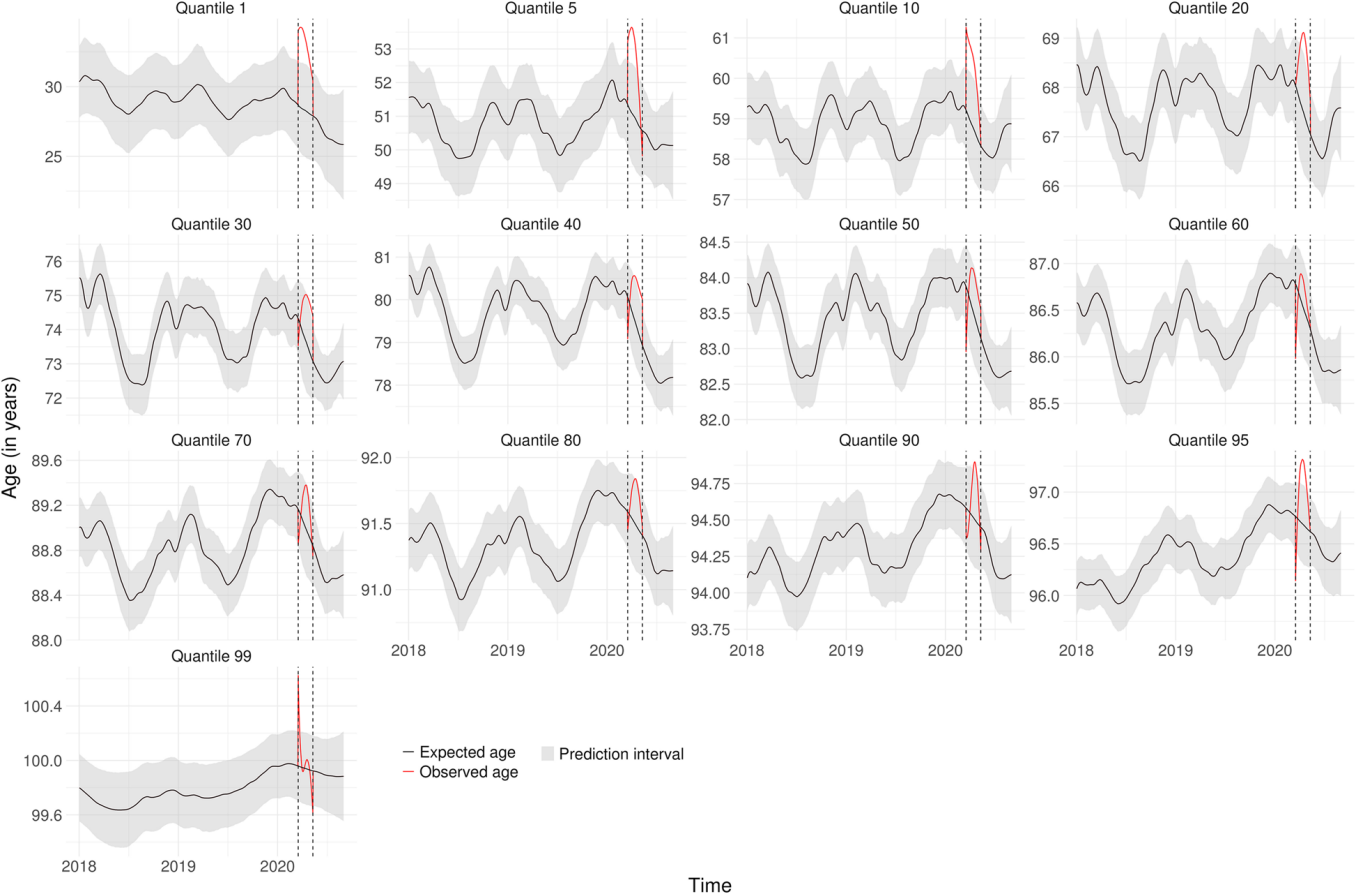
Expected age at death without lockdown and observed age with lockdown for each quantile in Bretagne

**Fig. 2:**
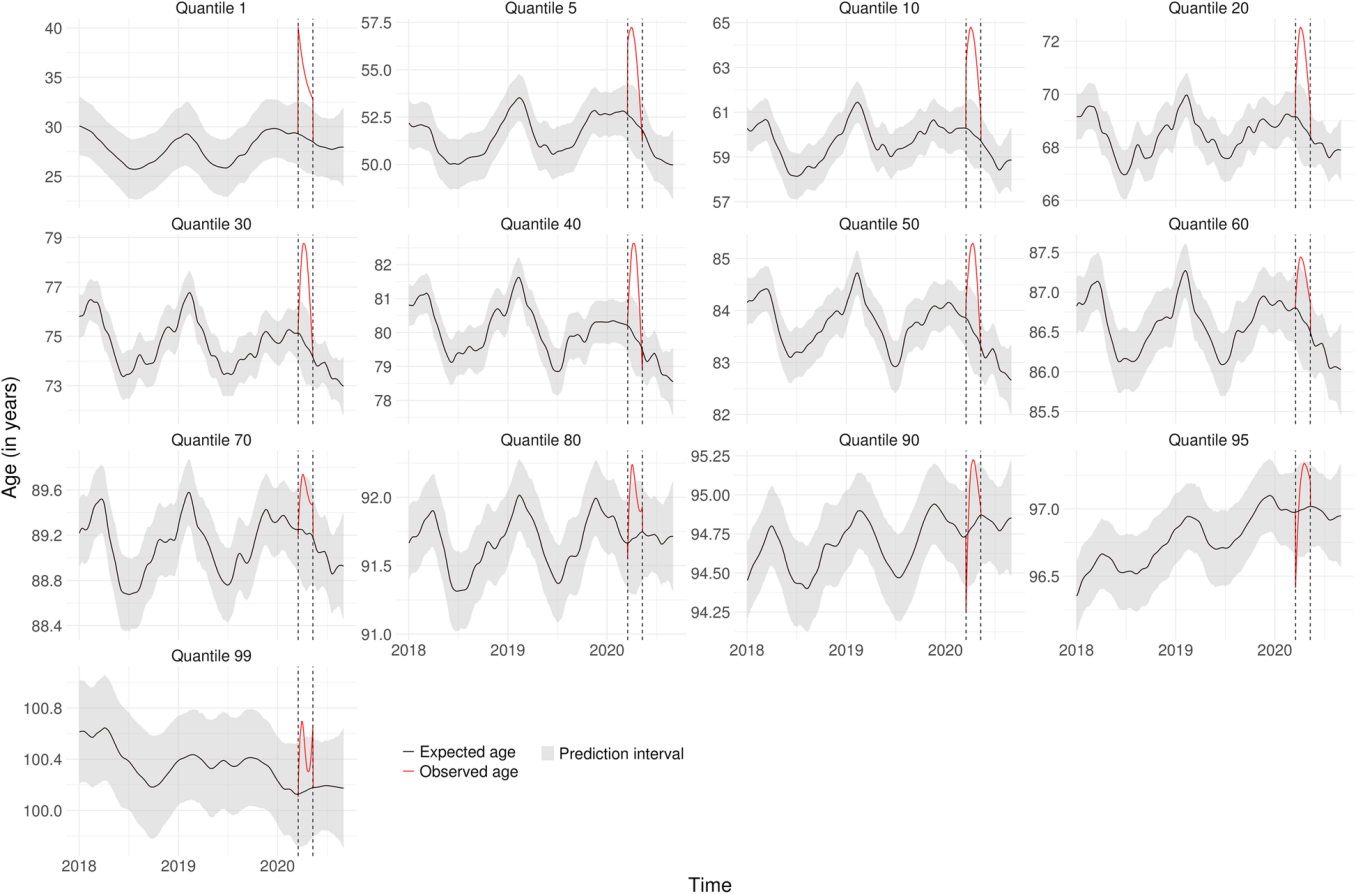
Expected age at death without lockdown and observed age with lockdown for each quantile in Bourgogne-Franche-Comté

**Fig. 3:**
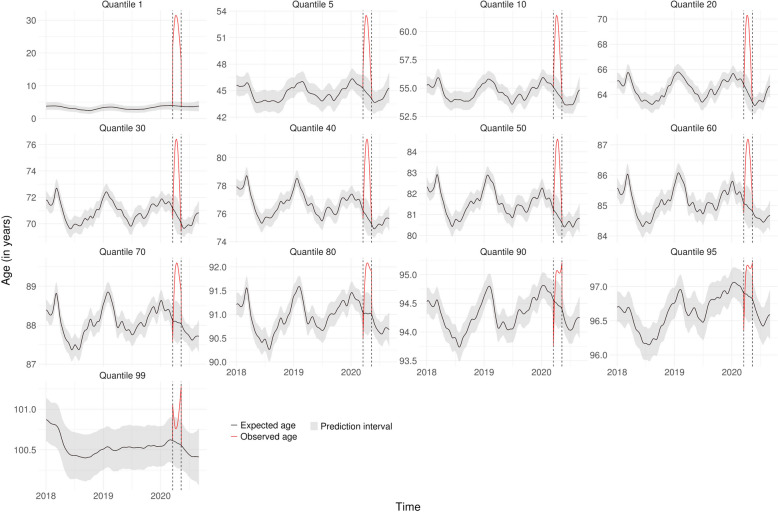
Expected age at death without lockdown and observed age with lockdown for each quantile in Ile-de-France

Figures 1, 2 and 3 show the lockdown effect on each quantile of age for the whole population in Bretagne, BFC and IDF, respectively. The corresponding figures for the 9 other regions are presented in Supplementary Fig. 1 to Fig. 9, Additional File 1, so as the observed frequencies of deaths by day and region for each quantile Supplementary Table 2 and Table 3, Additional File 1. The introduction of lockdown led to an immediate increase in the first quantiles of age at death, irrespective of the region, while the increase was more gradual for older age groups. Analysis of the highest quantiles showed an opposite effect in relation to the youngest, with an initial drop before returning to the usual level, even if we had some uncertainties around the estimates. In Bretagne, for example, we observed an immediate shift in age starting from the 1st quantile and declining up to the 40th, before vanishing. The situation in BFC and IDF was similar except that the shift did not seem to vanish with a more pronounced effect in IDF up to the 80th quantile.

**Fig. 4:**
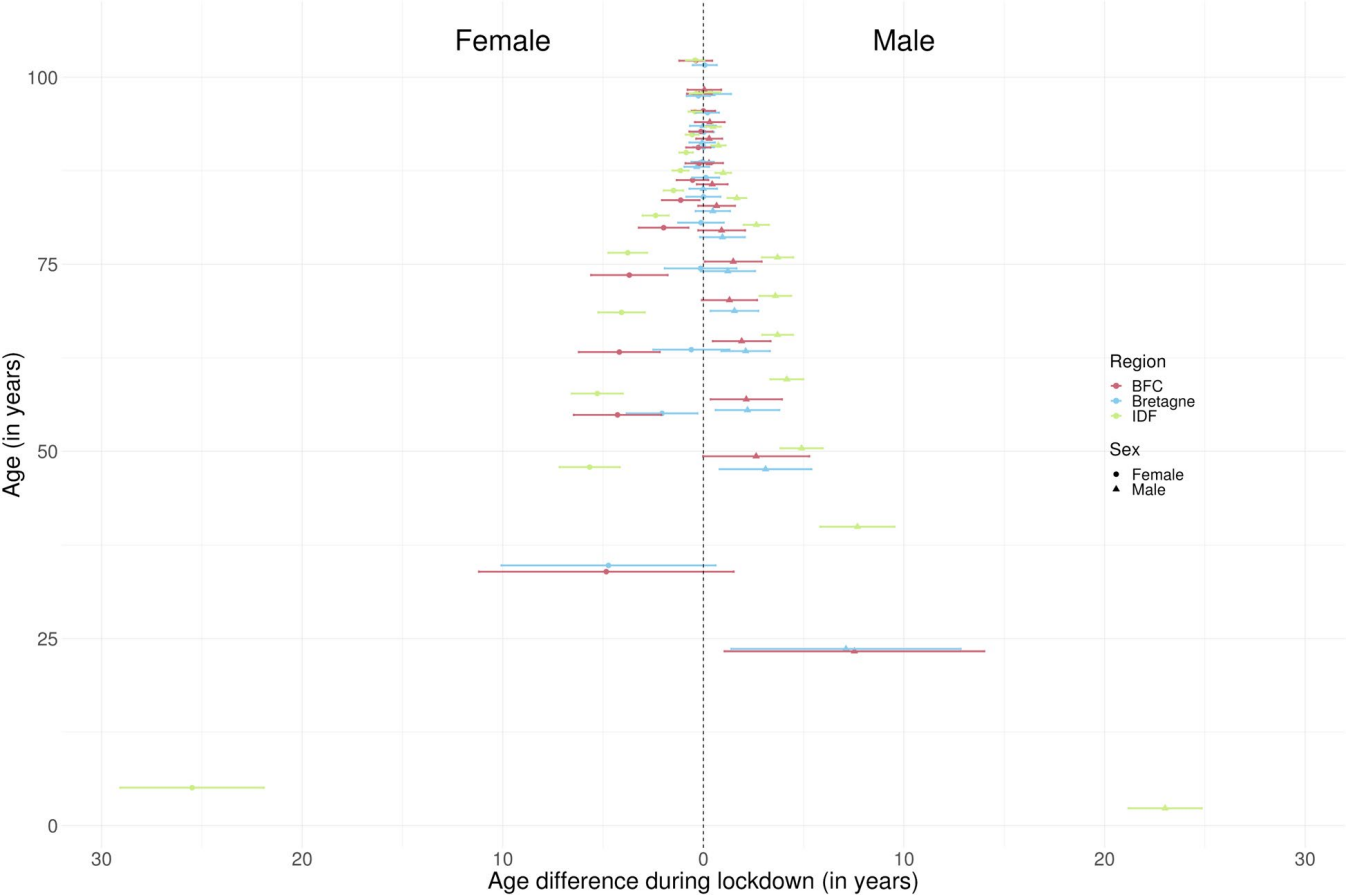
Average age difference during lockdown according to sex and region for the 13 quantiles analysed. Figure 4 legend: BFC: Bourgogne-Franche-Comté; IDF: Ile-de-France

Figure 4 Represents the average age difference during lockdown by sex in our 3 representative regions for the 13 quantiles analysed (full results for all regions are presented in Supplementary Table 4 and Table 5, Additional File 1). Each age quantile is positioned on the y-axis on the expected age without lockdown, to help the interpretation. For males, we observed a major impact on the first quantile with young men dying more than 7.1 [1.4–12.8] and 7.5 [1.1–14.0] years older during lockdown than expected in Bretagne and BFC, respectively. The median increase across all regions was equal to 7.3 years (significant in 10 out of the 12 regions) and the maximum was 23.0 [21.2–24.9] years in IDF. In Bretagne and BFC, this difference rapidly reduced from the 5th quantile onwards and decreased slightly up to age 75 (quantile 40); whereas in IDF, the increase of age at death for males was observed up to age 93 (quantile 95). On the opposite, the 1st quantile in females increased by 4.6 years in median, with heterogeneity between regions (significant in 4 regions out of the 12 regions). In Bretagne, only an increase in the 5th quantile of 2.1 [0.3–3.8] years could be highlighted in females during lockdown. In BFC, we observed a positive shift in age of about 4 years of the first quantiles (quantiles 5 and 10), which decreased slowly up to the 40th quantile corresponding to 85 years old. As for men, in IDF region, a shift was observed up to quantile 90 (around 95 years) with a significant one in the first quantile of around 25.5 [21.9–29.1] years. In Bretagne, a maximum quantile shift of 3.8 [1.3–5.8] points was observed for males at a constant age of 66 years, *i.e.* the quantile was 3.8 points lower at 66 years without lockdown, whereas no clear difference was detected for females (full results for all regions are presented in Supplementary Table 6 and Table 7, Additional File 1). On the contrary, in BFC, maximum quantile shifts of 5.7 [2.3–7.7] and 3.4 [0.5–5.6] were noticed at 77 years for females and 67 years for males, respectively. Quantile shift was maximum for IDF with an observed shift of 8.3 [6.6–9.7] and 7.3 [5.2–8.7] points for males and females at age 80, respectively.

**Fig. 5:**
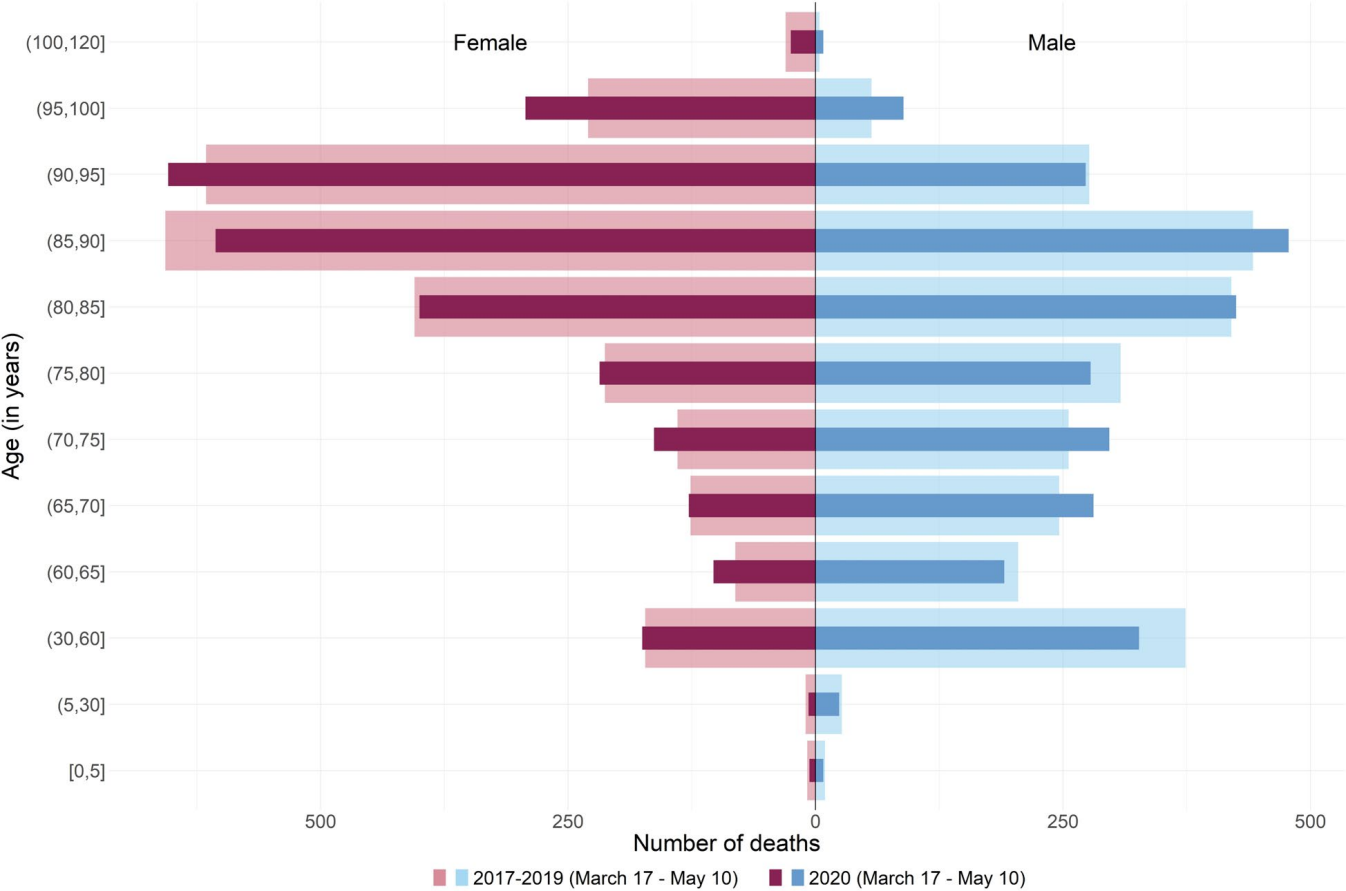
Evolution of the number of deaths according to age at death and sex during the lockdown in 2020 and the corresponding period in 2017–2019 in Bretagne. Figure 5 legend: Dark red and blue stand for 2020 and light red and blue stand for 2017–2019

**Fig. 6:**
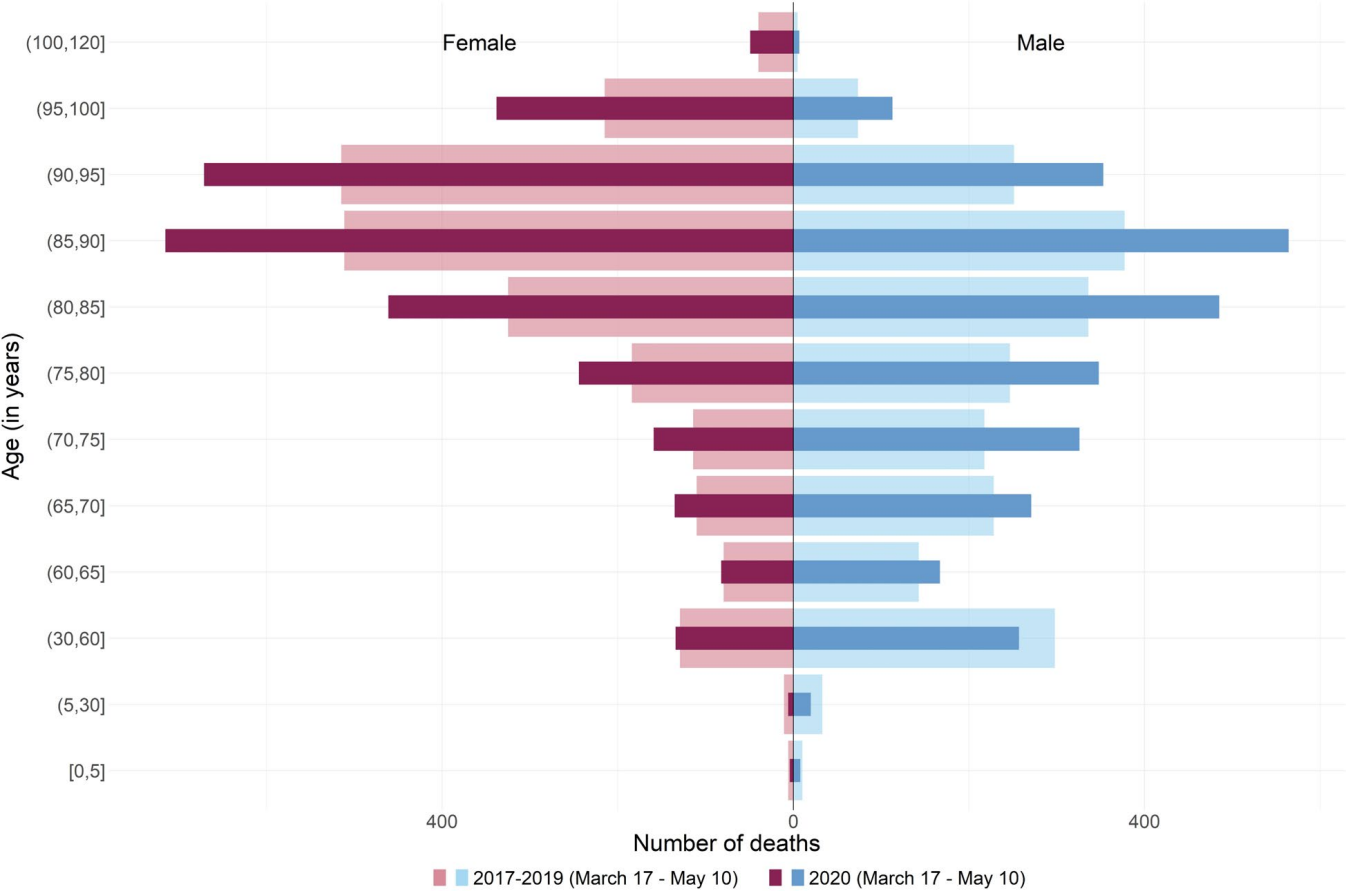
Evolution of the number of deaths according to age at death and sex during the lockdown in 2020 and the corresponding period in 2017–2019 in Bourgogne-Franche-Comté. Figure 6 legend: dark red and blue stand for 2020 and light red and blue stand for 2017–2019

**Fig. 7:**
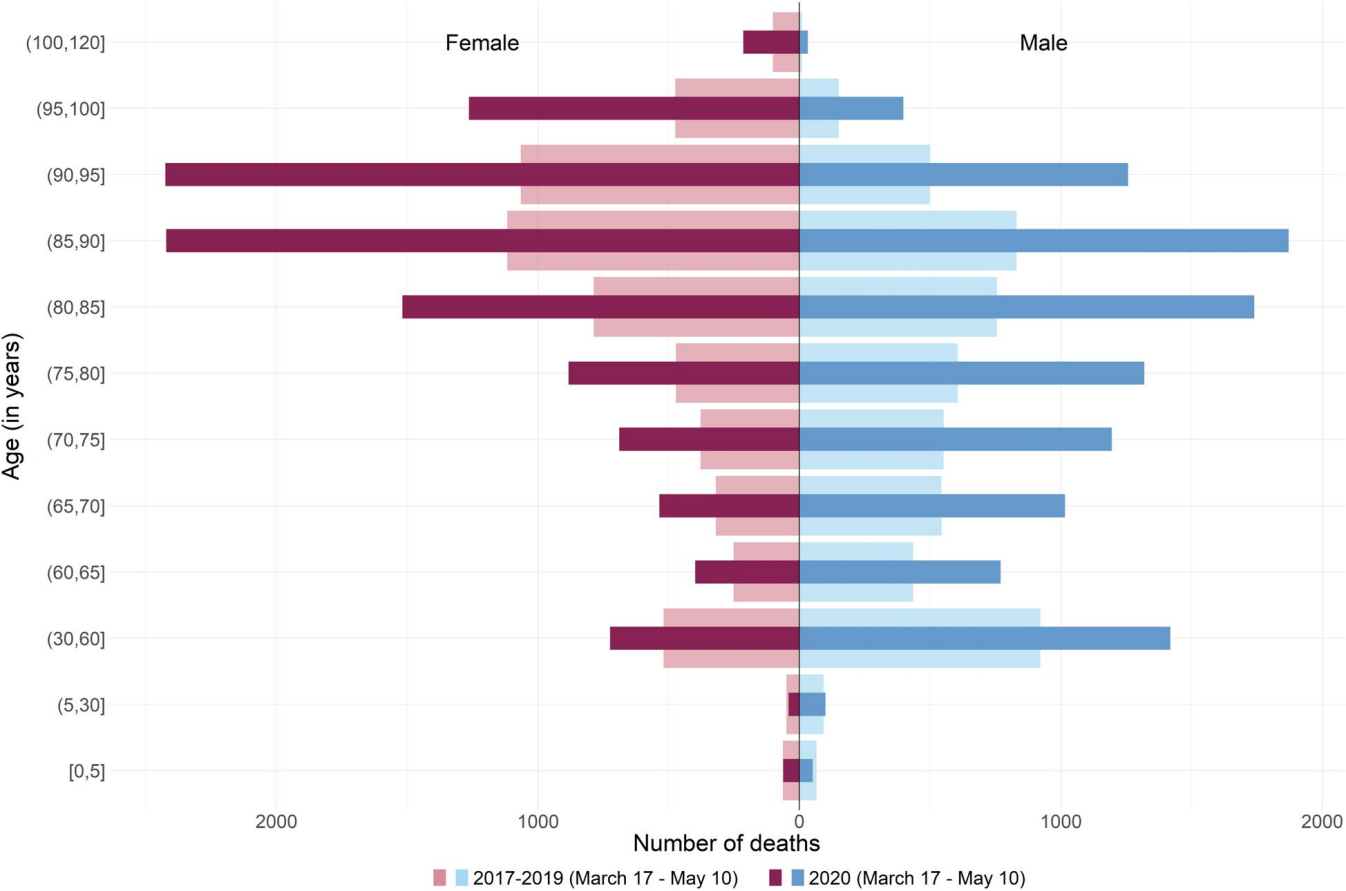
Evolution of the number of deaths according to age at death and sex during the lockdown in 2020 and the corresponding period in 2017–2019 in Ile-de-France. Figure 7 legend: dark red and blue stand for 2020 and light red and blue stand for 2017–2019

Quantiles are parameters that partition a set of values into subset of equal size. In that sense, a positive shift in age distribution can be related to fewer deaths than usual under the lowest quantiles and/or more deaths than usual for the highest ones. Examination of crude death pyramid allows us to determine which hypothesis is the most plausible (see Supplementary Fig. 10 to Fig. 18, Additional File 1, for the 9 other regions). In Bretagne, death pyramid showed fewer deaths than usual for the lowest quantiles, especially for men, and no change otherwise (Fig. 5). Deaths pyramid in BFC showed a different pattern with an overall excess mortality for males over 60 years old and females over 65 years old (Fig. 6). The region IDF showed the most important excess mortality across all regions, with an excess observed starting from the 30–60 years age group for females and even the 5–30 group for males (Fig. 7).

## Discussion

The Covid-19 pandemic has challenged health systems around the globe and led to changes in the care and detection of diseases but also on daily life, especially because of lockdowns. Compared to previous studies in the literature, this work focuses on an innovative outcome: the distribution of age at death. It analysed the lockdown effect in metropolitan French regions differently affected by Covid-19 through 3 representatives: IDF the most affected region, BFC severely affected and Bretagne which was preserved.

Our results showed the lockdown influenced the observed distributions of age at death quantiles in France. These excess or deficit of deaths, depending of regions, were driven by different age categories. Proportionally, there were less young people, mainly male, that died during the lockdown, with an increase in age of death at the first quantile around 7 years in median. This was equivalent to a positive shift of 1 point of quantile, *i.e.* the first quantile for age during lockdown being almost equal to the second one if there had been no lockdown. Looking at the raw comparison of deaths pyramids in Bretagne and BFC, this increase in age at death for males may be related up to 65 and 60 years old (almost quantile 20 and 10, respectively) to less deaths for these populations. Above this value, the shift vanished reflecting similar mortality distribution during lockdown compared to otherwise. In BFC, we may still postulate that the positive shift for the lowest quantile was also due to less deaths at younger ages, but also to an excess mortality for elderly people. This was confirmed by the shift in age deaths quantile showing two peaks at 67 (quantile 20) and 77 years (quantile 50) for males compared to only one peak at 66 (quantile 20) in Bretagne. The situation was less pronounced and vanished more quickly for females in Bretagne as the premature mortality is usually less pronounced than for male. For females in BFC, the positive shift in age distribution could mostly be related to an excess mortality over 80 years old. The positive shift stayed over 4 points of age between 74 and 84 (quantiles 20 and 40, respectively), indicating that most of the extra deaths occurred over these ages. The IDF region had a complete change in its distribution of age at death during lockdown because of the higher number of deaths across almost all age groups independently from sex, especially in elderly people. A positive shift over 4 points of age was observed between 61 (quantile 10) and 88 (quantile 70) years in females and between 53 and 86 years (quantile 10 and 80, respectively) in males. This can be explained by the density of hospital beds in IDF (18.2% of all beds in metropolitan France [26, 27]) and the high spread of the virus in this region, which has led to a quick overrun of intensive care unit capacities [28]. In the regions highly affected by Covid-19, the highest quantiles show a drop at the start of lockdown, followed by a return to expected, while analysis of the age distribution shows a general increase in mortality among the elderly. This suggests that the initial fall was driven by an increase in mortality at lower ages which far exceeded that at the oldest ages, before this proportion stabilised again.

Quantile regression on age at death makes it possible to study the change over the whole fine-grained mortality distribution without being influenced by the arbitrary choice of age classes, and this for each day of the lockdown and not just over the whole period. Age-specific mortality, as shown in Figs. 5, 6 and 7 reflects the rate or number of deaths in arbitrarily selected age groups over the entire period of lockdown. Changing the cut-off age group changes the reading and interpretation of this pyramid. In addition, age-specific mortality compares observed mortality with its past, averaged over several years or not. This crude comparison suffers from several pitfalls, as shown for example by Kepp et al. [29], in contrast to the interpolation performed in our analysis, as discussed in Faisant et al. [10]. Moreover, unlike traditional statistical methods such as time series modelling [30], quantile regression interpolates the distribution of quantiles during and in the absence of lockdown giving the background estimate rather than just forecasting estimates.

Our study provides a deeper insight into changes in the structure of age at death during lockdown due to Covid-19 and confirms the results outlined in previous studies. So as Aburto et al. [7], we highlighted a difference of age distribution between sexes regarding age at death. In addition, we showed the first quantiles of age at death increased during the lockdown period, which was in accordance with the older deaths observed also in UK compared to expected [31]. This non-mortality of young people will probably lead to a gain in life expectancy in the long term. The present study did not analyse causes of death, it is thus difficult to see an impact on mortality from a specific cause, but several hypotheses can be put forward to explain these changes. Firstly, the evolution of causes of death during lockdown may be an explanation. Mobility restrictions reduced traffic and air pollution [32], which had a direct impact on the number of road traffic accidents [4, 14]. In France, a 63%-decrease of road mortality during first lockdown in France compared to 2017–2019 had been observed [33]. Moreover, a reduction around 60% of trips according to mobile data was noticed in Bretagne and BFC during lockdown compared to the first week of February 2020 [34]. Younger persons and men being more at risk of road traffic accidents may have been preserved and may concur to the increase in the first quantiles of age at death. Indeed, 24% of road traffic fatalities concerned people aged 24 years and younger in France in 2020 and 78% of fatalities were men [35]. Moreover, a reduction in the number of deaths in public places in France which could be coherent with less traffic accident and related death during the lockdown has been observed [36] (also observed in the UK [37]). Largely, less traumatic events took place during lockdown due to restrictions and may also explain the changes in the first quantiles. This hypothesis seems to be confirmed by the immediate increase observed only in the first age quantiles. In addition, the decrease in the incidence of hospital admissions for selected pathologies described in Europe [2, 3] and in France [12, 13] indicated a change in the causes of death observed at hospital and may have also an impact on age at death.

Secondly, the heterogeneous evolution of the pandemic on the French territory could be considered worth mentioning. The first wave of Covid-19 spread mainly from east to west in France. BFC, a region in the east of the country, was one of the first to be hit by the virus and, because of its proximity to a major spreading event (religious gathering in a contiguous region at the beginning of the pandemic). All the regions were therefore not at the same stage of the epidemic when the lockdown began [28]. Moreover, the regions did not have the same hospitalisation rates and intensive care unit demands, with a better situation in Bretagne [28]. Older deaths observed in BFC and IDF could be explained by a greater spread of the virus and thus more deaths related to Covid-19 in older people, which may be in line with O’Donnel with an older age at death due to Covid-19 [31].

The data used in this study are exhaustive and were extracted in June 2023, allowing for a 3-year delay between the end of the study period and the study. Therefore, only a few deaths may be missing in both regions, and their small number may not affect the results. In addition, some deaths were dropped from the original dataset because of a missing characteristic (sex, age or region of death), corresponding to 47 510 deaths, or 0.8% of the total number of deaths available at the national level. These missing characteristics may be due to inconsistencies in the death certificate or missing data, but may not have influenced our results. Our dataset did not include any medical information or information on patients' comorbidities. We thus limited our analysis to age at death from all causes. This lack of clinical data meant that we were unable to assess the impact of covariates such as the presence or absence of comorbidities on changes in age at death, despite the fact that international studies have shown a change in patient profile, particularly concerning this parameter [38]. Moreover, we limited our study period to the first lockdown of the Covid-19 pandemic, while two other lockdowns occurred in France (in November 2020 and April 2021). The first lockdown is a case study in which the population as a whole was subject to extremely strict movement restrictions, combined with a virtual standstill in economic activity. This was much less the case during the other two periods, that could be considered as “soft lockdowns”. It would be interesting to study the distribution of age at death during these other two lockdowns to see if they had the same effect. Moreover, the variant circulating at the time of the study was the original strain (Wuhan strain); other variants (in particular Alpha, Delta and Omicron variants) circulated subsequently with different virulence and transmissibility. Changes in these parameters may also affect age at death and should be investigated.

## Conclusions

The present analysis shows that the first quantiles of age at death increased differentially according to sex during the lockdown period, and that overall shift seems to depend on prior epidemic intensity before lockdown. It complements previously published studies focusing on the number of deaths and excess mortality during lockdown. These results observed in France, may also be observed in other countries where lockdown had been set.

### Supplementary Information

**Additional File 1:** Content: Descriptive statistics of deaths by region (**Supplementary Table 1**), Observed frequencies of deaths by day, by region, by quantile and gender from January 2011 to August 2020 (**Supplementary Tables 2 and 3**), Expected age at death without lockdown, observed age at death during lockdown and average age difference during lockdown by region and gender for each quantile (**Supplementary Tables 4 and 5**), Difference in quantile value between lockdown and expected at a fixed age at death, by region and gender (**Supplementary Tables 6 and 7**), Expected age at death with and without lockdown effect for each quantile selected by region (**Supplementary Figs. 1 to 9**) and Evolution of the number of deaths according to age at death and sex during the lockdown in 2020 and the corresponding period in 2017–2019 by region (**Supplementary Figs. 10 to 18**).

## Data Availability

The data underlying this article are publicly available on the Insee website: https://www.insee.fr/fr/information/4190491.

## Abbreviations

SARS-CoV-2: Severe acute respiratory syndrome coronavirus 2
Covid-19: 2019 Coronavirus disease
BFC: Bourgogne-Franche-Comté
CVL: Centre-Val de Loire
IDF: Ile-de-France
Insee: French National Institute of Statistics and Economic Studies
QGAM: Quantile generalized additive model
