# Supplementary material for: Age at death during the Covid-19 lockdown in French metropolitan regions: a non parametric quantile regression approach
Source: BMC Public Health. 2024 May 7;24:1251. doi: 10.1186/s12889-024-18699-0 (PMC11075327; doi:10.1186/s12889-024-18699-0)
Supplement: Supplementary file 1 — Additional File 1: Content: Descriptive statistics of deaths by region (Supplementary Table 1), Observed frequencies of deaths by day, by region, by quantile and gender from January 2011 to August 2020 (Supplementary Tables 2 and 3), Expected age at death without lockdown, observed age at death during lockdown and average age difference during lockdown by region and gender for each quantile (Supplementary Tables 4 and 5), Difference in quantile value between lockdown and expected at a fixed age at death, by region and gender (Supplementary Tables 6 and 7), Expected age at death with and without lockdown effect for each quantile selected by region (Supplementary Figs. 1 to 9) and Evolution of the number of deaths according to age at death and sex during the lockdown in 2020 and the corresponding period in 2017–2019 by region (Supplementary Figs. 10 to 18). [file 12889_2024_18699_MOESM1_ESM.pdf]

Additional file 1

Title: Age at death during the Covid-19 lockdown in French metropolitan regions: A non parametric quantile regression approach

Supplementary Table 1 Descriptive statistics of deaths by region

Region	Number of deaths over the period	Average number of deaths per day over the period	Number of deaths during the lockdown	Average age at death over the period	Average age at death during the lockdown	Region population
Auvergne-Rhône-Alpes	643 440	182.3	12 776	78.7	81.2	8 078 652
Bourgogne-Franche-Comté	280 626	79.5	5917	79.0	81.4	2 801 695
Bretagne	328 240	93.0	5457	78.5	79.9	3 373 835
Centre-Val de Loire	244 097	69.2	4654	79.3	81.4	2 574 863
Grand-Est	499 764	141.6	12 781	77.7	80.6	5 562 651
Hauts-de-France	524 061	148.5	10 524	76.4	78.4	5 997 734
Ile-de-France	719 086	203.7	22 346	76.2	79.7	12 271 794
Normandie	312 537	88.5	5647	78.0	79.7	3 325 522
Nouvelle Aquitaine	606 952	171.9	9871	79.6	80.9	6 033 952
Occitanie	544 578	154.3	9279	79.3	80.4	5 973 969
Pays de la Loire	478 617	135.6	8400	79.1	81.0	3 832 120
Provence-Alpes-Côte d'Azur	321 906	91.2	5721	78.7	80.4	5 098 666

Study period: January 1st, 2011 to August 31st, 2020. Lockdown period: March 17th to May 11th, 2020.

Source of population data: Institut national de la statistique et des études économiques (Insee). Populations légales des régions en 2020 [Internet]. [cited 2024 Mar 4]. Available from: https://www.insee.fr/fr/statistiques/6683011?sommaire=6683037

Supplementary Table 2 Observed frequencies of deaths by day, by region, by quantile, from January 2011 to August 2020 - Males

	**Quantiles**												
	**1**	**5**	**10**	**20**	**30**	**40**	**50**	**60**	**70**	**80**	**90**	**95**	**99**
Auvergne-Rhône-Alpes	0.9	4.5	9.1	18.2	27.2	36.3	45.4	54.5	63.6	72.6	81.7	86.3	89.9
Bourgogne-Franche-Comté	0.4	2.0	4.0	8.0	12.0	15.9	19.9	23.9	27.9	31.9	35.9	37.9	39.5
Bretagne	0.5	2.3	4.6	9.3	13.9	18.5	23.2	27.8	32.5	37.1	41.7	44.1	45.9
Centre-Val de Loire	0.3	1.7	3.5	7.0	10.5	14.0	17.5	21.0	24.5	28.0	31.5	33.2	34.6
Grand-Est	0.7	3.5	7.0	14.1	21.1	28.2	35.2	42.2	49.3	56.3	63.3	66.9	69.7
Hauts-de-France	0.7	3.7	7.5	14.9	22.4	29.8	37.3	44.7	52.2	59.7	67.1	70.8	73.8
Ile-de-France	1.0	5.0	10.1	20.1	30.2	40.3	50.4	60.4	70.5	80.6	90.6	95.7	99.7
Normandie	0.9	4.3	8.6	17.2	25.8	34.4	43.1	51.7	60.3	68.9	77.5	81.8	85.2
Nouvelle Aquitaine	0.4	2.2	4.5	8.9	13.4	17.8	22.3	26.7	31.2	35.7	40.1	42.3	44.1
Occitanie	0.8	3.9	7.7	15.5	23.2	31.0	38.7	46.5	54.2	62.0	69.7	73.6	76.7
Pays de la Loire	0.7	3.4	6.7	13.4	20.1	26.9	33.6	40.3	47.0	53.7	60.4	63.8	66.5
Provence-Alpes-Côte d'Azur	0.5	2.3	4.6	9.3	13.9	18.5	23.1	27.8	32.4	37.0	41.7	44.0	45.8

Interpretation: In Auvergne-Rhône-Alpes, the quantile 1 for age at death in males corresponds to 0.9 death per day on average during the whole period.

Supplementary Table 3 Observed frequencies of deaths by day, by region, by quantile, from January 2011 to August 2020 - Females

	**Quantiles**												
	**1**	**5**	**10**	**20**	**30**	**40**	**50**	**60**	**70**	**80**	**90**	**95**	**99**
Auvergne-Rhône-Alpes	0.9	4.6	9.1	18.3	27.4	36.6	45.7	54.9	64.0	73.1	82.3	86.9	90.5
Bourgogne-Franche-Comté	0.4	2.0	4.0	7.9	11.9	15.9	19.8	23.8	27.7	31.7	35.7	37.6	39.2
Bretagne	0.5	2.3	4.7	9.3	14.0	18.6	23.3	28.0	32.6	37.3	41.9	44.3	46.1
Centre-Val de Loire	0.3	1.7	3.4	6.8	10.2	13.7	17.1	20.5	23.9	27.3	30.7	32.4	33.8
Grand-Est	0.7	3.6	7.1	14.2	21.3	28.5	35.6	42.7	49.8	56.9	64.0	67.6	70.4
Hauts-de-France	0.7	3.7	7.4	14.8	22.2	29.5	36.9	44.3	51.7	59.1	66.5	70.2	73.1
Ile-de-France	1.0	5.1	10.3	20.6	30.9	41.2	51.5	61.8	72.1	82.3	92.6	97.8	101.9
Normandie	0.9	4.3	8.6	17.2	25.7	34.3	42.9	51.5	60.0	68.6	77.2	81.5	84.9
Nouvelle Aquitaine	0.4	2.2	4.4	8.8	13.2	17.6	22.0	26.4	30.8	35.2	39.5	41.7	43.5
Occitanie	0.8	3.8	7.7	15.4	23.0	30.7	38.4	46.1	53.7	61.4	69.1	72.9	76.0
Pays de la Loire	0.7	3.4	6.8	13.7	20.5	27.4	34.2	41.1	47.9	54.7	61.6	65.0	67.7
Provence-Alpes-Côte d'Azur	0.4	2.2	4.5	9.0	13.5	18.0	22.4	26.9	31.4	35.9	40.4	42.6	44.4

Interpretation: In Auvergne-Rhône-Alpes, the quantile 1 for age at death in females corresponds to 0.9 death per day on average during the whole period.

Supplementary Table 4 Expected age at death without lockdown, observed age at death during lockdown and average age difference during lockdown by region for each quantile - Males

**Quantile**	**1**	**5**	**10**	**20**	**30**	**40**	**50**	**60**	**70**	**80**	**90**	**95**	**99**
Auvergne-Rhône-Alpes													
Expected age	19.0	45.5	54.4	63.2	69.1	74.4	78.8	82.3	85.2	88.1	91.3	93.6	97.8
Lockdown age	28.3	49.8	57.7	66.5	71.8	76.9	80.6	83.5	86.2	88.8	91.7	93.9	97.9
Difference	9.3	4.3	3.3	3.2	2.7	2.5	1.8	1.3	1.0	0.8	0.4	0.3	0.1
[95%CI]	[5.6; 13.1]	[2.5; 6.2]	[2.0; 4.5]	[2.3; 4.2]	[1.9; 3.6]	[1.6; 3.4]	[1.1; 2.5]	[0.7; 1.8]	[0.6; 1.5]	[0.4; 1.2]	[0.0; 0.7]	[-0.1; 0.7]	[-0.4; 0.6]
Bourgogne-Franche-Comté													
Expected age	23.3	49.3	56.9	64.7	70.2	75.4	79.5	82.8	85.7	88.5	91.8	94.0	98.3
Lockdown age	30.8	52.0	59.1	66.6	71.5	76.8	80.4	83.5	86.1	88.8	92.1	94.3	98.4
Difference	7.5	2.6	2.1	1.9	1.3	1.5	0.9	0.7	0.4	0.3	0.3	0.3	0.1
[95%CI]	[1.1; 14.0]	[0; 5.3.0]	[0.4; 3.9]	[0.5; 3.4]	[-0.1; 2.7]	[0.1; 2.9]	[-0.3; 2.1]	[-0.3; 1.6]	[-0.3; 1.2]	[-0.4; 1.0]	[-0.4; 0.9]	[-0.4; 1.1]	[-0.8; 0.9]
Bretagne													
Expected age	23.6	47.6	55.5	63.4	68.8	74.1	78.6	82.1	85.1	88.0	91.3	93.5	97.8
Lockdown age	30.7	50.7	57.7	65.5	70.3	75.3	79.6	82.6	85.1	87.7	91.2	93.5	98.2
Difference	7.1	3.1	2.2	2.1	1.6	1.2	1.0	0.5	0.0	-0.3	-0.1	0.0	0.4
[95%CI]	[1.4; 12.8]	[0.8; 5.4]	[0.6; 3.8]	[0.9; 3.3]	[0.4; 2.7]	[-0.1; 2.6]	[-0.2; 2.1]	[-0.4; 1.3]	[-0.7; 0.7]	[-1.0; 0.3]	[-0.7; 0.6]	[-0.7; 0.6]	[-0.5; 1.4]
Centre-Val de Loire													
Expected age	23.5	47.6	55.5	63.8	69.7	75.2	79.6	83.0	85.8	88.7	91.8	94.1	98.3
Lockdown age	33.3	51.5	57.8	65.4	72.0	77.6	81.2	83.9	86.7	89.4	92.1	93.9	98.1
Difference	9.8	3.9	2.2	1.6	2.3	2.4	1.6	1.0	0.8	0.7	0.3	-0.2	-0.2
[95%CI]	[5.0; 14.6]	[2.1; 5.8]	[0.7; 3.8]	[0.3; 3.0]	[1.0; 3.6]	[1.1; 3.7]	[0.6; 2.6]	[0.2; 1.8]	[0.2; 1.5]	[0.1; 1.3]	[-0.3; 0.8]	[-0.7; 0.3]	[-0.9; 0.6]
Grand-Est													
Expected age	20.4	45.5	54.0	62.1	67.6	72.8	77.4	81.0	84.0	86.9	90.3	92.6	96.9
Lockdown age	29.8	51.0	58.7	66.0	70.9	76.1	79.8	82.4	84.9	87.5	90.9	93.0	97.1
Difference	9.5	5.5	4.7	3.8	3.3	3.2	2.4	1.5	1.0	0.6	0.6	0.3	0.2
[95%CI]	[5.9; 13.0]	[3.7; 7.3]	[3.5; 5.9]	[3.0; 4.7]	[2.4; 4.1]	[2.3; 4.2]	[1.7; 3.2]	[0.9; 2.1]	[0.5; 1.4]	[0.2; 1.1]	[0.2; 1]	[-0.1; 0.7]	[-0.3; 0.7]
Hauts-de-France													
Expected age	20.4	44.0	52.2	59.9	65.0	69.8	74.9	79.3	82.9	86.2	89.8	92.3	96.5
Lockdown age	24.5	45.8	53.4	61.1	66.3	71.2	76.8	80.7	83.8	86.8	90.1	92.7	96.4
Difference	4.1	1.8	1.3	1.2	1.3	1.4	1.9	1.4	0.9	0.6	0.3	0.4	-0.1
[95%CI]	[0.9; 7.2]	[0.0; 3.5]	[0.1; 2.4]	[0.4; 2.1]	[0.5; 2.1]	[0.5; 2.3]	[0.9; 2.9]	[0.6; 2.2]	[0.3; 1.5]	[0.1; 1.1]	[-0.2; 0.8]	[-0.1; 0.9]	[-0.6; 0.5]

95%CI: 95% credibility interval.

Supplementary Table 4 Expected age at death without lockdown, observed age at death during lockdown and average age difference during lockdown by region for each quantile - Males (continued)

**Quantile**	**1**	**5**	**10**	**20**	**30**	**40**	**50**	**60**	**70**	**80**	**90**	**95**	**99**
Ile-de-France													
Expected age	2.3	40.0	50.4	59.6	65.6	70.8	75.9	80.3	83.9	87.2	90.9	93.4	98.0
Lockdown age	25.3	47.6	55.3	63.8	69.3	74.4	79.6	82.9	85.5	88.2	91.6	93.9	98.3
Difference	23.0	7.7	4.9	4.2	3.7	3.6	3.7	2.6	1.7	1.0	0.8	0.5	0.4
[95%CI]	[21.2; 24.9]	[5.8; 9.5]	[3.8; 6.0]	[3.3; 5.0]	[2.9; 4.5]	[2.8; 4.4]	[2.9; 4.5]	[2.0; 3.3]	[1.2; 2.1]	[0.6; 1.4]	[0.4; 1.1]	[0.1; 0.9]	[-0.1; 0.8]
Normandie													
Expected age	21.7	45.2	53.4	61.6	67.2	72.5	77.5	81.3	84.5	87.5	90.9	93.2	97.4
Lockdown age	30.9	48.1	55.0	63.4	68.3	73.2	78.1	82.0	84.7	87.6	90.8	93.2	97.4
Difference	9.2	2.8	1.6	1.8	1.1	0.7	0.6	0.6	0.3	0.1	-0.1	0.0	0.0
[95%CI]	[3.9; 14.4]	[1.1; 4.6]	[0.4; 2.9]	[0.7; 2.9]	[0.1; 2.2]	[-0.5; 1.9]	[-0.5; 1.7]	[-0.2; 1.4]	[-0.4; 0.9]	[-0.4; 0.7]	[-0.6; 0.5]	[-0.6; 0.5]	[-0.7; 0.8]
Nouvelle Aquitaine													
Expected age	24.2	47.8	55.8	64.2	70.2	75.7	79.9	83.2	86.0	88.7	91.8	94.0	98.1
Lockdown age	26.3	48.9	57.5	65.1	70.8	76.5	80.7	83.7	86.3	88.9	91.8	94.0	98.2
Difference	2.1	1.1	1.7	0.9	0.6	0.8	0.7	0.5	0.4	0.2	0.0	0.0	0.0
[95%CI]	[-3.0; 7.1]	[-0.7; 2.9]	[0.4; 2.9]	[-0.1; 1.9]	[-0.3; 1.6]	[-0.1; 1.8]	[0.0; 1.5]	[-0.1; 1.1]	[-0.1; 0.8]	[-0.2; 0.6]	[-0.4; 0.4]	[-0.4; 0.4]	[-0.6; 0.7]
Occitanie													
Expected age	22.2	46.8	55.3	64.1	70.1	75.6	79.8	83.1	85.9	88.7	91.8	94.0	98.2
Lockdown age	28.8	49.3	56.4	64.9	70.7	75.9	80.2	83.4	86.3	89.1	91.9	94.1	98.1
Difference	6.6	2.5	1.2	0.8	0.6	0.3	0.3	0.3	0.4	0.4	0.1	0.1	-0.1
[95%CI]	[2.2; 11.0]	[0.5; 4.4]	[-0.2; 2.5]	[-0.3; 1.9]	[-0.5; 1.6]	[-0.6; 1.3]	[-0.5; 1.2]	[-0.4; 0.9]	[-0.1; 0.9]	[-0.1; 0.8]	[-0.3; 0.5]	[-0.3; 0.6]	[-0.6; 0.4]
Pays de la Loire													
Expected age	20.5	45.5	53.9	62.5	68.4	74.1	78.8	82.4	85.4	88.3	91.5	93.7	98.1
Lockdown age	24.4	47.2	55.5	64.2	69.7	75.5	79.8	83.0	85.6	88.4	91.5	93.6	98.2
Difference	3.9	1.7	1.6	1.7	1.3	1.4	1.0	0.6	0.3	0.1	0.0	-0.2	0.1
[95%CI]	[-0.7; 8.5]	[-0.3; 3.7]	[0.0; 3.2]	[0.5; 2.8]	[0.2; 2.4]	[0.1; 2.6]	[0.0; 2.0]	[-0.2; 1.3]	[-0.4; 0.9]	[-0.4; 0.6]	[-0.5; 0.6]	[-0.6; 0.3]	[-0.7; 0.9]
Provence-Alpes-Côte d'Azur													
Expected age	20.5	46.3	55.0	64.0	69.9	75.1	79.4	82.7	85.6	88.4	91.7	94.0	98.3
Lockdown age	27.6	49.1	56.9	66.1	71.7	76.4	80.0	83.1	85.9	88.6	91.9	94.4	98.4
Difference	7.1	2.9	1.9	2.1	1.8	1.3	0.6	0.4	0.3	0.2	0.2	0.5	0.1
[95%CI]	[3.1; 11.0]	[0.9; 4.9]	[0.4; 3.5]	[0.9; 3.2]	[0.8; 2.8]	[0.3; 2.3]	[-0.2; 1.4]	[-0.3; 1.1]	[-0.2; 0.9]	[-0.2; 0.7]	[-0.2; 0.7]	[0.0; 0.9]	[-0.4; 0.7]

95%CI: 95% credibility interval.

Supplementary Table 5 Expected age at death without lockdown, observed age at death during lockdown and average age difference during lockdown by region for each quantile - Females

**Quantile**	**1**	**5**	**10**	**20**	**30**	**40**	**50**	**60**	**70**	**80**	**90**	**95**	**99**
Auvergne-Rhône-Alpes													
Expected age	28.4	54.1	63.4	74.2	80.3	83.8	86.4	88.6	90.6	92.6	95.4	97.7	101.9
Lockdown age	39.7	57.8	66.2	76.1	81.9	84.7	86.9	88.9	90.8	92.9	95.6	97.7	101.8
Difference	11.2	3.6	2.8	1.9	1.6	0.9	0.5	0.3	0.3	0.3	0.2	0.0	-0.1
[95%CI]	[6.5; 16.0]	[1.9; 5.3]	[1.4; 4.3]	[0.8; 3.1]	[0.9; 2.3]	[0.4; 1.4]	[0.1; 0.9]	[-0.1; 0.7]	[0.0; 0.6]	[0.0; 0.6]	[-0.2; 0.5]	[-0.3; 0.3]	[-0.6; 0.4]
Bourgogne-Franche-Comté													
Expected age	33.9	54.9	63.3	73.6	79.9	83.6	86.2	88.5	90.6	92.7	95.5	97.8	102.2
Lockdown age	38.8	59.1	67.5	77.2	81.9	84.7	86.8	88.7	90.9	92.9	95.5	98.0	102.6
Difference	4.8	4.3	4.2	3.7	2.0	1.1	0.5	0.2	0.3	0.1	0.0	0.2	0.4
[95%CI]	[-1.5; 11.2]	[2.1; 6.5]	[2.2; 6.2]	[1.8; 5.6]	[0.7; 3.2]	[0.2; 2.1]	[-0.3; 1.3]	[-0.5; 0.9]	[-0.4; 0.9]	[-0.5; 0.7]	[-0.6; 0.6]	[-0.4; 0.8]	[-0.4; 1.2]
Bretagne													
Expected age	34.8	55.1	63.6	74.4	80.6	84.0	86.6	88.7	90.7	92.7	95.3	97.5	101.6
Lockdown age	39.5	57.1	64.2	74.6	80.7	84.0	86.5	88.7	90.6	92.6	95.1	97.8	101.6
Difference	4.7	2.1	0.6	0.1	0.1	0.0	-0.1	0.0	0.0	0.0	-0.2	0.3	-0.1
[95%CI]	[-0.6; 10.1]	[0.3; 3.8]	[-1.3; 2.5]	[-1.7; 1.9]	[-1.0; 1.3]	[-0.9; 0.9]	[-0.8; 0.6]	[-0.5; 0.6]	[-0.5; 0.5]	[-0.5; 0.5]	[-0.8; 0.4]	[-0.3; 0.8]	[-0.7; 0.5]
Centre-Val de Loire													
Expected age	34.1	55.0	63.8	74.5	80.6	84.1	86.6	88.8	90.9	92.9	95.8	98.1	102.3
Lockdown age	41.4	56.6	66.3	76.6	82.1	85.0	87.3	89.4	91.1	93.0	96.1	98.5	103.2
Difference	7.3	1.6	2.5	2.0	1.4	0.9	0.7	0.6	0.3	0.1	0.4	0.4	1.0
[95%CI]	[1.5; 13.1]	[-0.4; 3.6]	[0.4; 4.6]	[0.3; 3.7]	[0.5; 2.4]	[0.2; 1.7]	[0.1; 1.3]	[0.1; 1.1]	[-0.1; 0.7]	[-0.3; 0.5]	[-0.1; 0.9]	[-0.1; 0.9]	[0.1; 1.8]
Grand-Est													
Expected age	31.6	53.5	61.8	72.3	78.9	82.6	85.3	87.5	89.6	91.8	94.4	96.8	101.0
Lockdown age	36.9	57.3	65.5	75.5	80.8	83.8	85.9	87.9	89.9	91.9	94.6	96.9	100.8
Difference	5.3	3.9	3.7	3.2	1.9	1.2	0.6	0.4	0.3	0.2	0.1	0.1	-0.2
[95%CI]	[1.4; 9.3]	[2.3; 5.4]	[2.4; 5.0]	[2.0; 4.3]	[1.1; 2.6]	[0.6; 1.7]	[0.2; 1.0]	[0.0; 0.7]	[-0.1; 0.6]	[-0.1; 0.5]	[-0.2; 0.5]	[-0.3; 0.6]	[-0.7; 0.3]
Hauts-de-France													
Expected age	30.2	52.2	60.2	70.4	77.8	81.9	84.7	87.0	89.2	91.4	94.0	96.4	100.7
Lockdown age	33.1	53.3	62.0	72.2	79.4	82.4	85.0	87.2	89.4	91.6	94.1	96.5	101.0
Difference	2.9	1.2	1.8	1.8	1.6	0.6	0.3	0.2	0.2	0.2	0.0	0.1	0.3
[95%CI]	[-1.6; 7.5]	[-0.4; 2.8]	[0.5; 3.0]	[0.5; 3.1]	[0.6; 2.6]	[-0.1; 1.2]	[-0.2; 0.8]	[-0.2; 0.6]	[-0.1; 0.6]	[-0.2; 0.5]	[-0.3; 0.3]	[-0.3; 0.4]	[-0.2; 0.8]

95%CI: 95% credibility interval.

Supplementary Table 5 Expected age at death without lockdown, observed age at death during lockdown and average age difference during lockdown by region for each quantile - Females (continued)

**Quantile**	**1**	**5**	**10**	**20**	**30**	**40**	**50**	**60**	**70**	**80**	**90**	**95**	**99**
Ile-de-France													
Expected age	5.1	47.9	57.7	68.6	76.5	81.5	84.9	87.5	89.9	92.3	95.4	97.8	102.3
Lockdown age	30.6	53.6	63.0	72.6	80.3	83.9	86.4	88.7	90.8	92.9	95.8	98.2	102.7
Difference	25.5	5.7	5.3	4.1	3.8	2.4	1.5	1.1	0.9	0.6	0.4	0.4	0.4
[95%CI]	[21.9; 29.1]	[4.2; 7.2]	[4.0; 6.6]	[2.9; 5.2]	[2.8; 4.7]	[1.7; 3.0]	[1.0; 2.0]	[0.7; 1.5]	[0.5; 1.2]	[0.2; 0.9]	[0.1; 0.7]	[0.0; 0.7]	[-0.1; 0.9]
Normandie													
Expected age	33.2	53.7	62.2	73.0	79.6	83.3	86.0	88.2	90.3	92.4	95.2	97.5	101.7
Lockdown age	36.7	54.8	62.0	72.9	79.6	83.3	85.9	88.2	90.3	92.4	95.1	97.5	101.5
Difference	3.5	1.1	-0.2	0.0	-0.1	0.0	-0.1	0.0	-0.1	0.0	-0.1	0.0	-0.2
[95%CI]	[-1.7; 8.7]	[-0.9; 3.0]	[-2.0; 1.5]	[-1.8; 1.7]	[-1.2; 1.0]	[-0.8; 0.7]	[-0.7; 0.5]	[-0.5; 0.5]	[-0.5; 0.4]	[-0.4; 0.4]	[-0.4; 0.3]	[-0.4; 0.5]	[-1.0; 0.6]
Nouvelle Aquitaine													
Expected age	36.5	55.6	64.1	75.0	81.0	84.4	86.9	89.0	91.0	93.0	95.8	98.1	102.3
Lockdown age	40.5	57.7	65.9	76.1	81.8	84.9	87.3	89.3	91.2	93.2	95.9	98.0	102.5
Difference	4.0	2.1	1.7	1.1	0.8	0.5	0.4	0.3	0.3	0.2	0.1	-0.1	0.3
[95%CI]	[-1.1; 9.2]	[0.4; 3.8]	[0.3; 3.2]	[-0.2; 2.4]	[0.0; 1.6]	[-0.1; 1.0]	[-0.1; 0.8]	[0.0; 0.7]	[-0.1; 0.6]	[-0.1; 0.5]	[-0.3; 0.5]	[-0.5; 0.3]	[-0.4; 0.9]
Occitanie													
Expected age	32.9	54.6	63.5	74.4	80.6	84.2	86.7	88.8	90.8	92.8	95.6	97.8	102.0
Lockdown age	36.5	54.7	64.1	75.2	81.2	84.4	86.7	88.8	90.9	93.0	95.7	97.9	102.0
Difference	3.6	0.0	0.6	0.8	0.5	0.2	0.0	0.0	0.1	0.1	0.1	0.0	0.0
[95%CI]	[-1.1; 8.4]	[-1.9; 1.9]	[-0.9; 2.1]	[-0.6; 2.1]	[-0.3; 1.4]	[-0.4; 0.8]	[-0.5; 0.5]	[-0.5; 0.4]	[-0.3; 0.4]	[-0.2; 0.5]	[-0.3; 0.6]	[-0.3; 0.3]	[-0.6; 0.7]
Pays de la Loire													
Expected age	32.1	54.3	63.3	74.4	80.6	84.1	86.7	88.8	90.8	92.8	95.7	98.0	102.1
Lockdown age	35.6	56.4	65.3	75.4	81.4	84.4	86.7	88.7	90.7	92.6	95.4	97.9	102.7
Difference	3.5	2.2	2.0	1.0	0.8	0.3	0.0	-0.1	-0.1	-0.3	-0.3	-0.1	0.5
[95%CI]	[-1.7; 8.7]	[-0.3; 4.6]	[0.1; 3.9]	[-0.6; 2.7]	[-0.1; 1.8]	[-0.4; 1.0]	[-0.5; 0.5]	[-0.6; 0.4]	[-0.5; 0.3]	[-0.7; 0.1]	[-0.7; 0.1]	[-0.6; 0.3]	[-0.4; 1.4]
Provence-Alpes-Côte d'Azur													
Expected age	31.5	54.6	63.7	74.0	80.2	83.9	86.5	88.7	90.8	92.9	95.7	98.0	102.2
Lockdown age	35.8	56.2	65.5	75.1	80.6	83.9	86.6	88.6	90.6	92.7	95.6	98.1	102.6
Difference	4.4	1.5	1.7	1.1	0.4	0.1	0.1	-0.2	-0.2	-0.1	-0.1	0.0	0.4
[95%CI]	[-0.2; 9.0]	[-0.5; 3.6]	[0.2; 3.3]	[-0.2; 2.4]	[-0.5; 1.3]	[-0.5; 0.7]	[-0.4; 0.5]	[-0.6; 0.3]	[-0.6; 0.2]	[-0.5; 0.2]	[-0.5; 0.3]	[-0.4; 0.5]	[-0.3; 1.2]

95%CI: 95% credibility interval.

Supplementary Table 6 Difference in quantile value between lockdown and expected at a fixed age at death, by region – Males

Age	Quantile difference [95%Credibility Interval]							
	Auvergne-Rhône-Alpes	*Bourgogne-Franche-Comté*	*Bretagne*	*Centre-Val de Loire*	*Grand-Est*	*Hauts-de-France*	*Ile-de-France*	*Nouvelle Aquitaine*
40	+1.0 [+0.5; +1.6]	+0.8 [+0.1; +1.9]	+0.9 [+0.2; +1.9]	+1.3 [+0.6; +2.3]	+1.2 [+0.7; +1.8]	+0.4 [+0.0; +0.8]	+1.4 [+1.1; +1.7]	+0.2 [–0.2; +0.9]
41	+1.0 [+0.5; +1.5]	+0.8 [+0.1; +1.9]	+0.8 [+0.2; +1.8]	+1.2 [+0.5; +2.2]	+1.2 [+0.7; +1.7]	+0.4 [+0.0; +0.8]	+1.7 [+1.4; +2.0]	+0.2 [–0.2; +0.8]
42	+0.9 [+0.5; +1.4]	+0.8 [+0.0; +1.8]	+0.8 [+0.2; +1.7]	+1.2 [+0.5; +2.1]	+1.1 [+0.7; +1.7]	+0.4 [+0.0; +0.8]	+2.0 [+1.7; +2.3]	+0.2 [–0.2; +0.8]
43	+0.9 [+0.5; +1.4]	+0.7 [+0.0; +1.7]	+0.8 [+0.2; +1.6]	+1.1 [+0.5; +2.0]	+1.1 [+0.7; +1.6]	+0.3 [+0.0; +0.7]	+2.3 [+2.0; +2.6]	+0.2 [–0.2; +0.8]
44	+0.9 [+0.5; +1.3]	+0.7 [+0.0; +1.6]	+0.7 [+0.2; +1.6]	+1.1 [+0.5; +1.9]	+1.1 [+0.7; +1.6]	+0.3 [–0.0; +0.7]	+2.6 [+2.3; +2.9]	+0.2 [–0.2; +0.7]
45	+0.8 [+0.4; +1.3]	+0.7 [+0.0; +1.6]	+0.7 [+0.1; +1.5]	+1.0 [+0.5; +1.8]	+1.1 [+0.7; +1.5]	+0.8 [–0.0; +1.1]	+2.9 [+2.6; +3.2]	+0.2 [–0.2; +0.7]
46	+1.0 [+0.6; +1.4]	+0.6 [+0.0; +1.5]	+0.7 [+0.1; +1.4]	+1.0 [+0.4; +1.7]	+1.2 [+0.9; +1.7]	+1.1 [+0.0; +1.5]	+3.2 [+2.8; +3.5]	+0.2 [–0.1; +0.7]
47	+1.4 [+1.0; +1.8]	+0.6 [+0.0; +1.4]	+0.6 [+0.1; +1.3]	+0.9 [+0.4; +1.6]	+1.6 [+1.3; +2.1]	+1.0 [+0.0; +1.9]	+3.5 [+2.7; +3.8]	+0.2 [–0.1; +0.6]
48	+1.7 [+1.4; +2.1]	+0.5 [+0.0; +1.3]	+0.8 [+0.3; +1.4]	+1.0 [+0.6; +1.7]	+2.0 [+1.7; +2.5]	+1.0 [+0.0; +2.1]	+3.6 [+2.5; +4.1]	+0.3 [–0.4; +0.7]
49	+2.1 [+1.4; +2.5]	+0.5 [–0.0; +1.2]	+1.2 [+0.5; +1.8]	+1.5 [+1.0; +2.1]	+2.4 [+2.1; +2.8]	+0.9 [+0.0; +2.0]	+3.4 [+2.4; +4.4]	+0.7 [–0.3; +1.1]
50	+2.4 [+1.3; +2.9]	+0.8 [+0.0; +1.5]	+1.6 [+0.5; +2.2]	+1.9 [+1.2; +2.4]	+2.8 [+2.2; +3.2]	+0.9 [+0.0; +1.9]	+3.3 [+2.3; +4.4]	+0.7 [–0.2; +1.5]
51	+2.3 [+1.3; +3.2]	+1.3 [+0.0; +1.9]	+1.9 [+0.5; +2.6]	+2.3 [+1.1; +2.8]	+3.2 [+2.2; +3.6]	+0.9 [+0.0; +1.8]	+3.5 [+2.6; +4.6]	+0.8 [–0.1; +1.8]
52	+2.3 [+1.3; +3.4]	+1.7 [+0.1; +2.4]	+1.9 [+0.5; +3.0]	+2.4 [+1.0; +3.1]	+3.2 [+2.1; +4.0]	+0.8 [+0.0; +1.7]	+3.9 [+3.1; +4.9]	+0.8 [–0.1; +1.8]
53	+2.2 [+1.2; +3.3]	+1.7 [+0.1; +2.8]	+1.8 [+0.4; +3.4]	+2.2 [+0.8; +3.5]	+3.1 [+2.1; +4.2]	+1.4 [+0.1; +2.2]	+4.3 [+3.6; +5.3]	+0.8 [+0.0; +1.8]
54	+2.1 [+1.2; +3.2]	+1.6 [+0.1; +3.2]	+1.7 [+0.4; +3.3]	+2.0 [+0.7; +3.5]	+3.1 [+2.1; +4.1]	+1.6 [+0.2; +2.8]	+4.8 [+4.0; +5.6]	+0.9 [+0.1; +1.8]
55	+2.4 [+1.5; +3.4]	+1.6 [+0.2; +3.4]	+1.6 [+0.4; +3.1]	+1.9 [+0.6; +3.3]	+3.6 [+2.7; +4.7]	+1.6 [+0.2; +3.1]	+5.2 [+4.1; +6.0]	+0.9 [+0.2; +1.8]
56	+2.9 [+2.0; +3.8]	+1.5 [+0.2; +3.3]	+1.9 [+0.7; +3.3]	+2.0 [+0.7; +3.3]	+4.2 [+3.4; +5.2]	+1.6 [+0.3; +3.1]	+5.2 [+4.1; +6.3]	+1.1 [+0.3; +1.9]
57	+3.4 [+2.3; +4.3]	+1.5 [+0.3; +3.2]	+2.4 [+0.8; +3.7]	+2.4 [+0.7; +3.7]	+4.8 [+4.0; +5.7]	+1.6 [+0.3; +3.0]	+5.2 [+4.0; +6.4]	+1.7 [+0.4; +2.5]
58	+3.7 [+2.3; +4.7]	+2.1 [+0.5; +3.6]	+2.8 [+0.9; +4.2]	+2.6 [+0.7; +4.1]	+5.4 [+4.2; +6.2]	+1.6 [+0.4; +2.9]	+5.1 [+3.9; +6.3]	+1.9 [+0.3; +3.1]
59	+3.7 [+2.3; +5.2]	+2.7 [+0.5; +4.1]	+2.8 [+0.9; +4.7]	+2.5 [+0.6; +4.4]	+5.7 [+4.2; +6.8]	+1.6 [+0.4; +2.9]	+5.0 [+3.9; +6.1]	+1.8 [+0.3; +3.4]
60	+3.7 [+2.4; +5.1]	+2.7 [+0.5; +4.6]	+2.8 [+0.9; +4.8]	+2.5 [+0.6; +4.5]	+5.6 [+4.1; +7.2]	+1.7 [+0.6; +2.9]	+5.1 [+4.0; +6.2]	+1.7 [+0.2; +3.2]
61	+3.7 [+2.4; +5.1]	+2.7 [+0.5; +5.0]	+2.7 [+1.0; +4.7]	+2.4 [+0.5; +4.3]	+5.4 [+4.0; +7.0]	+2.3 [+0.8; +3.5]	+5.6 [+4.6; +6.7]	+1.5 [+0.1; +3.1]
62	+3.7 [+2.4; +5.0]	+2.6 [+0.5; +4.9]	+2.7 [+1.0; +4.6]	+2.3 [+0.4; +4.2]	+5.3 [+3.9; +6.8]	+2.4 [+0.8; +4.0]	+6.1 [+5.1; +7.2]	+1.4 [+0.0; +2.9]
63	+3.7 [+2.5; +5.0]	+2.6 [+0.6; +4.8]	+2.7 [+1.1; +4.5]	+2.2 [+0.4; +4.1]	+5.7 [+4.3; +7.1]	+2.4 [+0.8; +4.0]	+6.6 [+5.6; +7.6]	+1.3 [–0.1; +2.8]
64	+4.1 [+3.0; +5.4]	+2.6 [+0.6; +4.7]	+3.1 [+1.5; +4.8]	+2.2 [+0.4; +4.0]	+6.1 [+4.9; +7.5]	+2.4 [+0.8; +4.0]	+7.0 [+5.5; +8.1]	+1.2 [–0.2; +2.6]
65	+4.7 [+3.6; +5.9]	+2.7 [+0.8; +4.8]	+3.6 [+1.5; +5.3]	+2.6 [+0.6; +4.4]	+6.6 [+5.4; +7.9]	+2.4 [+0.9; +4.0]	+6.8 [+5.4; +8.3]	+1.4 [–0.3; +2.8]
66	+5.3 [+3.8; +6.4]	+3.2 [+0.7; +5.2]	+3.8 [+1.3; +5.8]	+2.8 [+0.8; +4.7]	+7.0 [+5.3; +8.3]	+2.6 [+1.0; +4.1]	+6.8 [+5.3; +8.3]	+1.4 [–0.3; +3.1]
67	+5.4 [+3.7; +7.0]	+3.4 [+0.5; +5.6]	+3.6 [+1.1; +6.1]	+3.0 [+1.0; +5.1]	+6.8 [+5.1; +8.6]	+2.7 [+1.0; +4.3]	+6.9 [+5.5; +8.3]	+1.3 [–0.4; +3.1]
68	+5.3 [+3.5; +7.1]	+3.2 [+0.3; +6.1]	+3.4 [+0.9; +5.9]	+3.2 [+1.2; +5.2]	+6.6 [+4.9; +8.4]	+2.7 [+1.0; +4.4]	+7.0 [+5.6; +8.4]	+1.3 [–0.4; +3.0]
69	+5.1 [+3.4; +6.9]	+2.9 [+0.1; +5.9]	+3.2 [+0.7; +5.7]	+3.4 [+1.3; +5.4]	+6.5 [+4.8; +8.3]	+2.8 [+1.1; +4.5]	+7.1 [+5.6; +8.5]	+1.2 [–0.5; +2.9]
70	+5.1 [+3.4; +6.9]	+2.7 [–0.1; +5.6]	+3.0 [+0.5; +5.5]	+3.6 [+1.6; +5.6]	+6.4 [+4.7; +8.1]	+2.8 [+1.1; +4.6]	+7.1 [+5.6; +8.6]	+1.1 [–0.6; +2.8]

Supplementary Table 6 Difference in quantile value between lockdown and expected at a fixed age at death, by region - Males (continued)

Age	Quantile difference [95%Credibility Interval]							
	Auvergne-Rhône-Alpes	*Bourgogne-Franche-Comté*	*Bretagne*	*Centre-Val de Loire*	*Grand-Est*	*Hauts-de-France*	*Ile-de-France*	*Nouvelle Aquitaine*
71	+5.1 [+3.5; +6.8]	+2.6 [–0.1; +5.5]	+2.8 [+0.3; +5.3]	+3.8 [+1.8; +5.9]	+6.2 [+4.6; +8.0]	+2.7 [+1.1; +4.5]	+7.0 [+5.5; +8.6]	+1.2 [–0.5; +2.9]
72	+5.1 [+3.4; +6.8]	+2.6 [–0.1; +5.3]	+2.7 [+0.1; +5.1]	+4.1 [+1.8; +6.1]	+6.2 [+4.6; +7.9]	+2.9 [+1.3; +4.5]	+7.0 [+5.5; +8.6]	+1.2 [–0.4; +2.9]
73	+5.0 [+3.3; +6.8]	+2.6 [+0.0; +5.2]	+2.6 [–0.1; +5.1]	+4.2 [+1.8; +6.4]	+6.3 [+4.6; +7.9]	+3.0 [+1.4; +4.6]	+7.0 [+5.5; +8.5]	+1.3 [–0.4; +3.0]
74	+4.9 [+3.2; +6.7]	+2.7 [+0.1; +5.3]	+2.5 [–0.3; +5.0]	+4.2 [+1.9; +6.5]	+6.5 [+4.8; +8.2]	+3.2 [+1.6; +4.8]	+7.0 [+5.5; +8.5]	+1.4 [–0.3; +3.0]
75	+5.1 [+3.3; +6.8]	+2.8 [+0.1; +5.4]	+2.6 [–0.3; +5.2]	+4.2 [+1.9; +6.6]	+6.8 [+5.0; +8.5]	+3.4 [+1.7; +5.1]	+7.0 [+5.5; +8.5]	+1.4 [–0.2; +3.1]
76	+5.3 [+3.6; +7.1]	+3.1 [+0.0; +5.8]	+2.6 [–0.3; +5.5]	+4.6 [+2.3; +7.0]	+7.0 [+4.8; +8.8]	+3.9 [+2.1; +5.6]	+7.1 [+5.6; +8.6]	+1.6 [–0.2; +3.3]
77	+5.5 [+3.3; +7.4]	+3.5 [–0.2; +6.3]	+2.5 [–0.4; +5.6]	+5.1 [+2.3; +7.5]	+6.6 [+4.4; +9.0]	+4.2 [+1.9; +6.1]	+7.5 [+6.0; +9.0]	+1.9 [–0.2; +3.9]
78	+5.1 [+2.9; +7.4]	+3.1 [–0.4; +6.8]	+2.3 [–0.4; +5.3]	+5.3 [+1.9; +8.0]	+6.5 [+4.4; +8.8]	+3.9 [+1.7; +6.4]	+7.9 [+6.4; +9.4]	+1.8 [–0.2; +4.1]
79	+4.7 [+2.7; +7.0]	+2.7 [–0.6; +6.5]	+2.4 [–0.6; +5.3]	+4.7 [+1.6; +8.5]	+6.7 [+4.7; +8.9]	+3.7 [+1.6; +6.0]	+8.3 [+6.6; +9.7]	+1.8 [–0.1; +3.9]
80	+5.0 [+3.0; +7.1]	+2.7 [–0.8; +6.2]	+2.5 [–0.8; +5.7]	+4.5 [+1.6; +7.9]	+6.7 [+4.0; +8.9]	+3.7 [+1.7; +5.9]	+8.2 [+6.0; +10.1]	+1.8 [–0.1; +3.7]
81	+4.9 [+2.7; +7.2]	+2.6 [–0.8; +6.3]	+2.1 [–1.0; +5.7]	+4.6 [+1.4; +7.8]	+5.7 [+3.2; +8.4]	+3.8 [+1.5; +6.0]	+7.8 [+5.8; +10.1]	+2.1 [–0.1; +4.3]
82	+4.4 [+2.3; +6.7]	+2.4 [–0.8; +6.1]	+1.6 [–1.4; +4.9]	+4.1 [+1.0; +7.7]	+5.1 [+2.9; +7.6]	+3.4 [+1.2; +5.8]	+7.6 [+5.6; +9.7]	+1.9 [–0.1; +4.3]
83	+4.3 [+2.3; +6.4]	+2.2 [–0.9; +5.7]	+1.3 [–1.8; +4.6]	+3.5 [+0.7; +6.9]	+4.5 [+2.4; +6.8]	+2.9 [+1.0; +5.1]	+7.3 [+5.0; +9.3]	+1.8 [–0.2; +3.9]
84	+4.1 [+2.2; +6.2]	+2.1 [–1.0; +5.5]	+0.7 [–2.2; +3.9]	+3.4 [+0.7; +6.4]	+3.8 [+1.8; +6.0]	+2.7 [+0.8; +4.7]	+6.3 [+4.2; +8.6]	+1.8 [–0.2; +3.9]
85	+3.9 [+2.1; +5.8]	+1.9 [–1.1; +5.2]	+0.0 [–2.6; +3.0]	+3.2 [+0.7; +6.0]	+3.2 [+1.4; +5.2]	+2.3 [+0.5; +4.2]	+5.4 [+3.6; +7.5]	+1.6 [–0.3; +3.6]
86	+3.7 [+2.0; +5.5]	+1.6 [–1.2; +4.7]	–0.4 [–2.9; +2.2]	+3.1 [+0.6; +5.7]	+2.8 [+1.1; +4.6]	+2.0 [+0.3; +3.8]	+4.6 [+3.0; +6.3]	+1.4 [–0.3; +3.3]
87	+3.3 [+1.7; +5.1]	+1.4 [–1.4; +4.2]	–0.8 [–3.2; +1.7]	+2.9 [+0.6; +5.4]	+2.3 [+0.7; +4.0]	+1.6 [+0.1; +3.2]	+3.8 [+2.3; +5.5]	+1.2 [–0.5; +3.0]
88	+3.0 [+1.3; +4.7]	+1.1 [–1.5; +3.9]	–0.9 [–2.7; +1.2]	+2.8 [+0.5; +5.2]	+1.8 [+0.6; +3.1]	+1.4 [–0.1; +2.8]	+2.9 [+1.6; +4.4]	+1.0 [–0.7; +2.7]
89	+2.3 [+0.8; +3.9]	+0.8 [–1.2; +3.4]	–0.7 [–2.5; +1.1]	+2.5 [+0.3; +4.8]	+1.8 [+0.5; +3.1]	+1.1 [–0.3; +2.6]	+2.6 [+1.4; +3.7]	+0.7 [–0.8; +2.2]
90	+1.8 [+0.4; +3.3]	+0.9 [–1.2; +3.0]	–0.5 [–2.3; +1.3]	+2.0 [–0.1; +4.2]	+1.7 [+0.5; +3.0]	+0.8 [–0.3; +2.2]	+2.4 [+1.3; +3.5]	+0.4 [–1.0; +1.8]
91	+1.4 [+0.0; +2.8]	+0.9 [–1.1; +2.9]	–0.2 [–1.8; +1.6]	+1.4 [–0.6; +3.6]	+1.2 [+0.2; +2.3]	+0.7 [–0.2; +1.6]	+2.1 [+1.0; +3.2]	+0.2 [–1.3; +1.6]
92	+0.7 [–0.1; +1.7]	+0.7 [–0.8; +2.7]	–0.1 [–1.5; +1.3]	+0.7 [–0.8; +2.7]	+1.0 [–0.0; +1.9]	+0.8 [–0.1; +1.7]	+1.4 [+0.6; +2.2]	–0.0 [–1.0; +1.2]
93	+0.7 [–0.3; +1.6]	+0.7 [–0.9; +2.2]	–0.1 [–1.3; +1.4]	+0.0 [–1.4; +1.5]	+0.3 [–0.1; +1.2]	+0.4 [–0.2; +1.0]	+1.2 [+0.3; +2.0]	–0.0 [–1.0; +0.9]
94	+0.3 [–0.2; +1.0]	+0.7 [–0.4; +2.3]	+0.0 [–0.6; +0.8]	–0.3 [–0.8; +0.9]	+0.3 [–0.1; +0.7]	+0.2 [–0.3; +0.7]	+0.4 [+0.1; +1.1]	–0.0 [–0.4; +0.9]
95	+0.2 [–0.2; +0.6]	+0.3 [–0.5; +1.0]	+0.1 [–0.6; +0.7]	–0.2 [–0.8; +0.3]	+0.3 [–0.2; +0.7]	+0.1 [–0.4; +0.6]	+0.4 [+0.0; +0.8]	–0.0 [–0.5; +0.4]
96	+0.2 [–0.3; +0.6]	+0.2 [–0.6; +0.9]	+0.2 [–0.5; +0.9]	–0.2 [–0.8; +0.4]	+0.2 [–0.3; +0.7]	-	+0.4 [–0.0; +0.7]	+0.0 [–0.5; +0.5]
97	+0.1 [–0.3; +0.6]	+0.1 [–0.7; +0.9]	+0.3 [–0.5; +1.0]	–0.2 [–0.8; +0.4]	-	-	+0.3 [–0.1; +0.7]	+0.0 [–0.5; +0.5]
98	-	-	-	-	-	-	-	-
99	-	-	-	-	-	-	-	-
100	-	-	-	-	-	-	-	-

Supplementary Table 6 Difference in quantile value between lockdown and expected at a fixed age at death, by region - Males (continued)

	Quantile difference [95%Credibility Interval]			
**Age**	*Normandie*	*Occitanie*	*Provence-Alpes-Côte d'Azur*	*Pays de la Loire*
40	+1.0 [+0.3; +2.0]	+0.7 [+0.2; +1.4]	+0.7 [+0.2; +1.3]	+0.4 [–0.1; +0.9]
41	+0.9 [+0.3; +1.9]	+0.7 [+0.2; +1.3]	+0.7 [+0.2; +1.2]	+0.4 [–0.1; +0.9]
42	+0.9 [+0.3; +1.7]	+0.6 [+0.2; +1.3]	+0.7 [+0.2; +1.2]	+0.4 [–0.1; +0.9]
43	+0.8 [+0.3; +1.6]	+0.6 [+0.1; +1.2]	+0.6 [+0.2; +1.1]	+0.3 [–0.1; +0.8]
44	+0.7 [+0.2; +1.5]	+0.6 [+0.1; +1.1]	+0.6 [+0.2; +1.1]	+0.3 [–0.0; +0.8]
45	+0.7 [+0.2; +1.4]	+0.5 [+0.1; +1.1]	+0.6 [+0.2; +1.1]	+0.3 [–0.0; +0.8]
46	+0.9 [+0.5; +1.6]	+0.5 [+0.1; +1.0]	+0.5 [+0.2; +1.0]	+0.5 [–0.2; +0.9]
47	+1.3 [+0.6; +1.9]	+0.5 [+0.2; +1.0]	+0.8 [+0.4; +1.3]	+0.9 [–0.1; +1.3]
48	+1.7 [+0.5; +2.2]	+0.9 [+0.3; +1.4]	+1.2 [+0.5; +1.6]	+1.0 [–0.1; +1.7]
49	+1.6 [+0.5; +2.5]	+1.3 [+0.2; +1.8]	+1.6 [+0.4; +2.0]	+1.0 [–0.1; +2.1]
50	+1.5 [+0.4; +2.8]	+1.4 [+0.2; +2.1]	+1.6 [+0.4; +2.4]	+1.0 [–0.1; +2.2]
51	+1.4 [+0.4; +2.6]	+1.3 [+0.1; +2.5]	+1.5 [+0.4; +2.7]	+1.0 [–0.1; +2.1]
52	+1.3 [+0.3; +2.4]	+1.2 [+0.0; +2.5]	+1.4 [+0.3; +2.7]	+1.0 [–0.0; +2.1]
53	+1.2 [+0.3; +2.3]	+1.1 [–0.0; +2.3]	+1.4 [+0.3; +2.6]	+1.0 [–0.0; +2.0]
54	+1.4 [+0.5; +2.5]	+0.9 [–0.1; +2.1]	+1.3 [+0.3; +2.5]	+1.0 [+0.0; +2.0]
55	+2.0 [+0.5; +2.9]	+0.8 [–0.1; +2.0]	+1.3 [+0.3; +2.4]	+1.5 [+0.1; +2.6]
56	+2.0 [+0.6; +3.4]	+1.1 [–0.3; +2.2]	+1.7 [+0.5; +2.8]	+1.8 [+0.1; +3.1]
57	+2.0 [+0.6; +3.5]	+1.3 [–0.3; +2.6]	+2.1 [+0.5; +3.2]	+1.9 [+0.2; +3.6]
58	+2.0 [+0.6; +3.5]	+1.2 [–0.3; +2.9]	+2.2 [+0.6; +3.7]	+1.9 [+0.3; +3.6]
59	+2.1 [+0.7; +3.5]	+1.2 [–0.3; +2.8]	+2.2 [+0.7; +3.8]	+1.9 [+0.3; +3.6]
60	+2.1 [+0.7; +3.5]	+1.2 [–0.3; +2.7]	+2.2 [+0.7; +3.8]	+1.9 [+0.4; +3.5]
61	+2.1 [+0.8; +3.5]	+1.1 [–0.3; +2.6]	+2.2 [+0.8; +3.8]	+1.9 [+0.5; +3.5]
62	+2.4 [+1.0; +3.7]	+1.1 [–0.3; +2.5]	+2.2 [+0.8; +3.7]	+1.9 [+0.6; +3.4]
63	+3.0 [+1.1; +4.3]	+1.0 [–0.3; +2.4]	+2.2 [+0.9; +3.7]	+2.2 [+0.9; +3.7]
64	+3.0 [+0.8; +4.9]	+1.0 [–0.4; +2.3]	+2.3 [+1.0; +3.7]	+2.7 [+0.8; +4.1]
65	+2.8 [+0.6; +5.0]	+1.3 [–0.5; +2.7]	+2.9 [+1.6; +4.2]	+2.8 [+0.7; +4.6]
66	+2.6 [+0.4; +4.8]	+1.3 [–0.6; +3.2]	+3.4 [+1.5; +4.8]	+2.6 [+0.6; +4.7]
67	+2.3 [+0.2; +4.5]	+1.2 [–0.6; +3.1]	+3.4 [+1.5; +5.3]	+2.5 [+0.5; +4.6]
68	+2.1 [–0.0; +4.3]	+1.1 [–0.7; +3.0]	+3.3 [+1.4; +5.3]	+2.4 [+0.4; +4.4]
69	+2.0 [–0.2; +4.1]	+1.1 [–0.7; +2.9]	+3.2 [+1.4; +5.2]	+2.3 [+0.3; +4.4]
70	+1.8 [–0.4; +4.0]	+1.0 [–0.8; +2.8]	+3.2 [+1.3; +5.1]	+2.3 [+0.3; +4.3]

Supplementary Table 6 Difference in quantile value between lockdown and expected at a fixed age at death, by region - Males (continued)

	**Quantile difference [95%Credibility Interval]**			
**Age**	*Normandie*	*Occitanie*	*Provence-Alpes-Côte d'Azur*	*Pays de la Loire*
71	+1.7 [–0.7; +3.9]	+1.0 [–0.9; +2.9]	+3.3 [+1.4; +5.2]	+2.3 [+0.3; +4.3]
72	+1.5 [–0.9; +3.8]	+0.9 [–1.0; +2.9]	+3.4 [+1.3; +5.3]	+2.3 [+0.3; +4.3]
73	+1.4 [–1.0; +3.7]	+0.8 [–1.0; +2.8]	+3.2 [+1.1; +5.3]	+2.3 [+0.3; +4.3]
74	+1.4 [–1.0; +3.8]	+0.8 [–1.1; +2.7]	+3.0 [+0.9; +5.1]	+2.4 [+0.2; +4.4]
75	+1.4 [–1.0; +3.8]	+0.7 [–1.2; +2.6]	+2.8 [+0.7; +4.9]	+2.7 [+0.2; +4.8]
76	+1.3 [–1.0; +3.7]	+0.8 [–1.4; +2.7]	+3.0 [+0.6; +5.1]	+2.8 [+0.2; +5.2]
77	+1.3 [–1.0; +3.6]	+0.8 [–1.3; +3.0]	+2.8 [+0.3; +5.3]	+2.6 [+0.1; +5.3]
78	+1.5 [–1.1; +3.9]	+0.8 [–1.2; +3.0]	+2.4 [–0.1; +5.1]	+2.4 [+0.0; +5.0]
79	+1.6 [–0.9; +4.4]	+0.8 [–1.2; +2.9]	+1.9 [–0.4; +4.5]	+2.3 [–0.1; +4.8]
80	+1.6 [–0.7; +4.4]	+0.9 [–1.4; +2.9]	+1.9 [–0.6; +4.3]	+2.7 [–0.2; +5.2]
81	+1.7 [–0.5; +4.2]	+0.9 [–1.3; +3.5]	+1.6 [–0.7; +4.3]	+2.3 [–0.3; +5.5]
82	+2.0 [–0.7; +4.3]	+0.9 [–1.2; +3.2]	+1.4 [–0.7; +3.9]	+2.0 [–0.4; +4.8]
83	+1.6 [–1.0; +4.4]	+0.8 [–1.2; +2.9]	+1.4 [–0.8; +3.6]	+1.9 [–0.7; +4.5]
84	+1.1 [–1.3; +3.8]	+1.0 [–1.0; +3.1]	+1.3 [–0.7; +3.6]	+1.5 [–0.9; +4.2]
85	+0.8 [–1.4; +3.2]	+1.2 [–0.7; +3.1]	+1.2 [–0.6; +3.3]	+1.1 [–1.2; +3.6]
86	+0.6 [–1.4; +2.8]	+1.3 [–0.4; +3.1]	+1.2 [–0.6; +3.1]	+0.8 [–1.3; +3.0]
87	+0.5 [–1.4; +2.6]	+1.4 [–0.3; +3.2]	+1.0 [–0.7; +2.8]	+0.6 [–1.4; +2.7]
88	+0.3 [–1.4; +2.1]	+1.4 [–0.3; +3.1]	+0.9 [–0.8; +2.6]	+0.4 [–1.5; +2.4]
89	+0.1 [–1.6; +1.9]	+1.3 [–0.3; +2.9]	+0.7 [–0.7; +2.1]	+0.3 [–1.4; +2.0]
90	–0.0 [–1.7; +1.7]	+1.0 [–0.6; +2.5]	+0.7 [–0.7; +2.1]	+0.2 [–1.5; +1.9]
91	–0.1 [–1.3; +1.4]	+0.7 [–0.8; +2.2]	+0.7 [–0.7; +2.1]	+0.1 [–1.6; +1.9]
92	–0.1 [–1.2; +1.0]	+0.3 [–0.7; +1.6]	+0.5 [–0.4; +1.8]	–0.0 [–1.2; +1.4]
93	–0.1 [–0.8; +1.1]	+0.3 [–0.7; +1.2]	+0.7 [–0.2; +1.6]	–0.2 [–1.3; +1.0]
94	–0.0 [–0.6; +0.5]	+0.2 [–0.3; +1.2]	+0.9 [–0.0; +1.8]	–0.1 [–0.6; +0.3]
95	–0.0 [–0.6; +0.5]	+0.1 [–0.4; +0.5]	+0.4 [–0.1; +0.9]	–0.1 [–0.6; +0.4]
96	+0.0 [–0.6; +0.6]	+0.0 [–0.5; +0.5]	+0.3 [–0.2; +0.8]	–0.0 [–0.6; +0.5]
97	-	–0.0 [–0.6; +0.5]	+0.2 [–0.3; +0.8]	+0.0 [–0.7; +0.6]
98	-	-	-	-
99	-	-	-	-
100	-	-	-	-

Supplementary Table 7 Difference in quantile value between lockdown and expected at a fixed age at death, by region - Females

	Quantile difference [95%Credibility Interval]							
**Age**	*Auvergne-Rhône-Alpes*	*Bourgogne-Franche-Comté*	*Bretagne*	*Centre-Val de Loire*	*Grand-Est*	*Hauts-de-France*	*Ile-de-France*	*Nouvelle Aquitaine*
40	-	-	-	-	-	+0.4 [–0.2; +1.3]	-	-
41	-	-	-	-	+0.9 [+0.3; +1.7]	+0.4 [–0.2; +1.2]	-	-
42	-	-	-	-	+0.9 [+0.3; +1.7]	+0.4 [–0.2; +1.2]	-	-
43	-	-	-	-	+0.9 [+0.3; +1.6]	+0.4 [–0.2; +1.1]	-	-
44	-	-	-	-	+0.9 [+0.3; +1.6]	+0.4 [–0.2; +1.1]	-	-
45	+1.4 [+0.7; +2.4]	-	-	-	+0.9 [+0.3; +1.5]	+0.3 [–0.1; +1.0]	+1.2 [+0.9; +1.7]	-
46	+1.3 [+0.6; +2.3]	+0.9 [+0.1; +2.1]	+0.7 [–0.0; +1.9]	-	+0.9 [+0.4; +1.5]	+0.3 [–0.1; +0.9]	+1.1 [+0.8; +1.6]	+0.7 [–0.1; +1.9]
47	+1.3 [+0.6; +2.2]	+0.9 [+0.1; +2.0]	+0.7 [–0.0; +1.8]	-	+0.8 [+0.4; +1.5]	+0.3 [–0.1; +0.9]	+1.1 [+0.7; +1.5]	+0.7 [–0.1; +1.8]
48	+1.2 [+0.6; +2.1]	+0.9 [+0.1; +2.0]	+0.7 [–0.0; +1.7]	+0.9 [+0.1; +2.4]	+0.8 [+0.4; +1.4]	+0.3 [–0.1; +0.8]	+1.0 [+0.7; +1.4]	+0.7 [–0.0; +1.7]
49	+1.1 [+0.5; +2.0]	+0.9 [+0.2; +1.9]	+0.6 [+0.0; +1.6]	+0.9 [+0.0; +2.2]	+0.8 [+0.4; +1.4]	+0.3 [–0.1; +0.8]	+1.3 [+1.0; +1.7]	+0.6 [–0.0; +1.6]
50	+1.1 [+0.5; +1.9]	+0.9 [+0.2; +1.9]	+0.6 [+0.0; +1.5]	+0.8 [+0.0; +2.1]	+0.8 [+0.4; +1.3]	+0.3 [–0.1; +0.7]	+1.7 [+1.4; +2.0]	+0.6 [–0.0; +1.6]
51	+1.0 [+0.5; +1.8]	+0.9 [+0.2; +1.8]	+0.6 [+0.0; +1.4]	+0.7 [–0.0; +1.9]	+0.8 [+0.4; +1.3]	+0.2 [–0.1; +0.7]	+2.0 [+1.7; +2.4]	+0.6 [+0.0; +1.5]
52	+0.9 [+0.4; +1.6]	+0.9 [+0.3; +1.8]	+0.6 [+0.0; +1.4]	+0.6 [–0.0; +1.7]	+0.8 [+0.4; +1.3]	+0.2 [–0.2; +0.6]	+2.4 [+2.1; +2.7]	+0.6 [+0.0; +1.4]
53	+0.9 [+0.4; +1.5]	+0.9 [+0.3; +1.7]	+0.5 [+0.0; +1.3]	+0.6 [–0.0; +1.6]	+0.8 [+0.4; +1.2]	+0.6 [–0.2; +1.0]	+2.7 [+2.1; +3.0]	+0.6 [+0.0; +1.3]
54	+0.8 [+0.4; +1.4]	+0.8 [+0.3; +1.6]	+0.5 [+0.0; +1.2]	+0.5 [–0.1; +1.4]	+1.0 [+0.6; +1.4]	+0.8 [–0.1; +1.4]	+2.9 [+2.1; +3.3]	+0.5 [+0.1; +1.2]
55	+1.1 [+0.7; +1.7]	+0.9 [+0.4; +1.6]	+0.5 [+0.1; +1.1]	+0.4 [–0.2; +1.2]	+1.4 [+1.1; +1.8]	+0.8 [–0.1; +1.7]	+2.9 [+2.1; +3.6]	+0.5 [+0.1; +1.2]
56	+1.4 [+1.0; +1.9]	+1.3 [+0.8; +2.0]	+0.8 [+0.1; +1.4]	+0.7 [–0.1; +1.5]	+1.8 [+1.4; +2.2]	+0.8 [–0.0; +1.7]	+2.8 [+2.1; +3.6]	+0.6 [+0.2; +1.2]
57	+1.7 [+1.0; +2.2]	+1.7 [+1.3; +2.3]	+1.2 [–0.1; +1.7]	+0.9 [–0.1; +1.7]	+2.2 [+1.4; +2.5]	+0.9 [+0.1; +1.8]	+2.8 [+2.1; +3.6]	+1.0 [+0.2; +1.5]
58	+1.9 [+1.0; +2.5]	+2.1 [+1.3; +2.7]	+1.1 [–0.2; +2.0]	+1.0 [–0.1; +1.9]	+2.3 [+1.4; +2.9]	+0.9 [+0.1; +1.8]	+2.9 [+2.2; +3.7]	+1.2 [+0.2; +1.8]
59	+1.9 [+0.9; +2.7]	+2.5 [+1.3; +3.0]	+1.0 [–0.3; +2.2]	+1.0 [–0.0; +2.0]	+2.3 [+1.4; +3.2]	+1.0 [+0.2; +1.8]	+3.3 [+2.6; +4.1]	+1.2 [+0.2; +2.1]
60	+1.8 [+0.9; +2.8]	+2.5 [+1.3; +3.4]	+0.9 [–0.5; +2.1]	+1.1 [+0.0; +2.1]	+2.3 [+1.4; +3.2]	+1.0 [+0.2; +1.8]	+3.7 [+3.0; +4.4]	+1.2 [+0.2; +2.2]
61	+1.8 [+0.9; +2.8]	+2.5 [+1.3; +3.7]	+0.7 [–0.6; +2.0]	+1.1 [+0.1; +2.2]	+2.3 [+1.4; +3.2]	+1.3 [+0.5; +2.1]	+4.1 [+3.4; +4.8]	+1.1 [+0.2; +2.2]
62	+1.7 [+0.8; +2.7]	+2.5 [+1.3; +3.8]	+0.6 [–0.7; +1.9]	+1.2 [+0.1; +2.2]	+2.4 [+1.5; +3.3]	+1.7 [+0.5; +2.5]	+4.5 [+3.7; +5.2]	+1.1 [+0.2; +2.1]
63	+1.7 [+0.8; +2.6]	+2.5 [+1.3; +3.8]	+0.5 [–1.0; +1.8]	+1.2 [+0.2; +2.3]	+2.7 [+1.9; +3.6]	+1.7 [+0.5; +2.9]	+4.9 [+3.6; +5.6]	+1.1 [+0.2; +2.1]
64	+1.9 [+1.0; +2.8]	+2.8 [+1.6; +4.1]	+0.5 [–1.2; +1.8]	+1.4 [+0.3; +2.4]	+3.0 [+2.2; +3.9]	+1.7 [+0.5; +3.0]	+4.8 [+3.5; +5.9]	+1.1 [+0.2; +2.0]
65	+2.2 [+1.3; +3.1]	+3.2 [+1.9; +4.4]	+0.5 [–1.3; +2.1]	+1.8 [+0.4; +2.8]	+3.4 [+2.2; +4.2]	+1.7 [+0.5; +3.0]	+4.7 [+3.4; +6.0]	+1.3 [+0.3; +2.3]
66	+2.5 [+1.2; +3.4]	+3.5 [+2.1; +4.8]	+0.5 [–1.3; +2.3]	+2.2 [+0.4; +3.3]	+3.5 [+2.2; +4.5]	+1.7 [+0.5; +3.0]	+4.5 [+3.3; +5.8]	+1.6 [+0.2; +2.5]
67	+2.6 [+1.1; +3.7]	+3.9 [+2.0; +5.1]	+0.4 [–1.3; +2.3]	+2.3 [+0.4; +3.7]	+3.4 [+2.2; +4.7]	+1.7 [+0.5; +3.0]	+4.4 [+3.2; +5.7]	+1.5 [+0.2; +2.8]
68	+2.5 [+1.1; +3.9]	+4.0 [+2.0; +5.5]	+0.4 [–1.4; +2.2]	+2.3 [+0.4; +4.1]	+3.4 [+2.1; +4.7]	+1.7 [+0.5; +3.0]	+4.3 [+3.1; +5.6]	+1.5 [+0.1; +2.9]
69	+2.4 [+1.0; +3.8]	+4.0 [+2.0; +5.9]	+0.4 [–1.4; +2.2]	+2.2 [+0.4; +4.2]	+3.3 [+2.1; +4.6]	+1.7 [+0.5; +3.0]	+4.3 [+3.1; +5.6]	+1.4 [+0.1; +2.8]
70	+2.3 [+1.0; +3.7]	+3.9 [+1.9; +6.0]	+0.3 [–1.4; +2.1]	+2.2 [+0.4; +4.1]	+3.3 [+2.1; +4.5]	+1.7 [+0.5; +3.0]	+4.6 [+3.3; +5.8]	+1.4 [+0.0; +2.7]

Supplementary Table 7 Difference in quantile value between lockdown and expected at a fixed age at death, by region - Females (continued)

	**Quantile difference [95%Credibility Interval]**							
**Age**	*Auvergne-Rhône-Alpes*	*Bourgogne-Franche-Comté*	*Bretagne*	*Centre-Val de Loire*	*Grand-Est*	*Hauts-de-France*	*Ile-de-France*	*Nouvelle Aquitaine*
71	+2.2 [+0.9; +3.6]	+3.9 [+1.9; +5.9]	+0.3 [–1.5; +2.1]	+2.1 [+0.3; +4.1]	+3.2 [+2.0; +4.5]	+1.9 [+0.7; +3.2]	+4.8 [+3.6; +6.0]	+1.3 [–0.0; +2.6]
72	+2.1 [+0.9; +3.5]	+3.8 [+1.9; +5.9]	+0.2 [–1.5; +2.0]	+2.1 [+0.3; +4.0]	+3.2 [+2.0; +4.4]	+2.3 [+0.7; +3.6]	+5.0 [+3.7; +6.2]	+1.2 [–0.1; +2.6]
73	+2.1 [+0.8; +3.4]	+3.8 [+1.8; +5.8]	+0.2 [–1.6; +1.9]	+2.0 [+0.3; +3.9]	+3.5 [+2.3; +4.7]	+2.4 [+0.7; +4.0]	+5.1 [+3.6; +6.4]	+1.2 [–0.1; +2.5]
74	+2.0 [+0.8; +3.3]	+4.0 [+2.1; +6.0]	+0.2 [–2.2; +1.9]	+2.0 [+0.3; +3.8]	+4.0 [+2.9; +5.2]	+2.3 [+0.7; +4.1]	+5.0 [+3.6; +6.6]	+1.1 [–0.2; +2.4]
75	+2.5 [+1.3; +3.7]	+4.6 [+2.6; +6.6]	+0.2 [–2.4; +2.2]	+2.3 [+0.6; +4.1]	+4.6 [+2.9; +5.7]	+2.3 [+0.7; +4.0]	+5.0 [+3.6; +6.5]	+1.1 [–0.3; +2.4]
76	+3.1 [+1.3; +4.3]	+5.1 [+2.6; +7.1]	+0.2 [–2.2; +2.9]	+3.0 [+0.6; +4.7]	+4.6 [+2.7; +6.2]	+2.3 [+0.7; +3.9]	+4.9 [+3.6; +6.4]	+1.7 [–0.3; +3.0]
77	+3.1 [+1.3; +4.9]	+5.7 [+2.3; +7.7]	+0.2 [–2.1; +3.0]	+3.3 [+0.6; +5.3]	+4.3 [+2.4; +6.4]	+2.2 [+0.8; +3.8]	+5.2 [+3.9; +6.7]	+1.7 [–0.2; +3.7]
78	+3.0 [+1.3; +4.9]	+5.4 [+2.0; +8.2]	+0.2 [–1.9; +2.8]	+3.1 [+0.7; +6.0]	+3.9 [+2.2; +5.9]	+2.4 [+1.0; +4.0]	+5.9 [+4.6; +7.3]	+1.6 [–0.2; +3.8]
79	+2.9 [+1.3; +4.7]	+4.8 [+1.7; +8.8]	+0.2 [–1.8; +2.6]	+2.9 [+0.7; +5.8]	+3.6 [+2.1; +5.4]	+3.5 [+1.1; +5.0]	+6.6 [+5.4; +8.0]	+1.6 [–0.1; +3.6]
80	+2.8 [+1.4; +4.4]	+4.3 [+1.5; +8.2]	+0.2 [–2.2; +2.4]	+2.7 [+0.7; +5.4]	+4.4 [+3.0; +6.1]	+3.3 [+0.6; +6.0]	+7.3 [+5.3; +8.7]	+1.5 [–0.1; +3.3]
81	+3.5 [+2.2; +5.0]	+4.9 [+1.8; +8.4]	+0.3 [–2.7; +2.8]	+3.0 [+1.2; +5.4]	+4.9 [+2.6; +6.7]	+2.5 [+0.2; +5.5]	+7.0 [+4.7; +9.3]	+1.4 [–0.0; +3.1]
82	+4.5 [+2.1; +6.0]	+5.3 [+1.4; +8.5]	+0.2 [–2.6; +3.6]	+4.1 [+1.1; +6.2]	+4.3 [+2.2; +6.7]	+1.8 [–0.3; +4.4]	+6.7 [+4.6; +9.2]	+2.3 [–0.1; +4.1]
83	+3.8 [+1.7; +6.3]	+4.5 [+0.9; +8.7]	+0.1 [–2.5; +3.2]	+3.7 [+0.8; +7.0]	+4.0 [+2.1; +6.1]	+1.8 [–0.4; +4.2]	+6.9 [+5.1; +9.2]	+2.0 [–0.2; +4.5]
84	+3.3 [+1.4; +5.5]	+4.1 [+0.5; +8.1]	–0.0 [–3.2; +2.8]	+3.2 [+0.6; +6.3]	+4.1 [+1.7; +6.3]	+1.4 [–0.6; +3.6]	+7.0 [+4.5; +9.1]	+1.6 [–0.3; +3.9]
85	+3.4 [+1.2; +5.5]	+3.9 [–0.2; +7.9]	–0.2 [–3.2; +3.3]	+3.6 [+0.6; +6.4]	+3.1 [+0.9; +5.5]	+1.3 [–0.8; +3.3]	+6.0 [+3.8; +8.5]	+1.8 [–0.3; +3.9]
86	+2.7 [+0.7; +4.9]	+2.9 [–0.9; +7.2]	–0.4 [–3.1; +2.7]	+3.2 [+0.5; +6.4]	+2.6 [+0.5; +4.8]	+1.1 [–0.8; +3.2]	+5.7 [+3.7; +7.9]	+1.7 [–0.3; +3.9]
87	+2.4 [+0.4; +4.4]	+2.2 [–1.6; +6.3]	–0.4 [–3.1; +2.6]	+3.1 [+0.5; +5.9]	+2.1 [+0.1; +4.2]	+1.0 [–0.9; +2.9]	+5.2 [+3.3; +7.3]	+1.7 [–0.2; +3.6]
88	+1.9 [–0.0; +3.9]	+1.5 [–2.1; +5.5]	–0.0 [–2.6; +2.7]	+3.0 [+0.4; +5.8]	+1.7 [–0.1; +3.5]	+1.0 [–0.8; +2.9]	+4.8 [+3.1; +6.8]	+1.7 [–0.2; +3.8]
89	+1.5 [–0.2; +3.4]	+1.0 [–2.0; +4.5]	+0.2 [–2.7; +2.9]	+2.9 [+0.2; +5.5]	+1.5 [–0.2; +3.2]	+1.1 [–0.6; +2.8]	+4.5 [+2.8; +6.4]	+1.6 [–0.2; +3.5]
90	+1.5 [–0.2; +3.2]	+1.1 [–1.8; +4.2]	+0.0 [–2.7; +2.9]	+2.4 [–0.2; +5.4]	+1.2 [–0.3; +2.9]	+1.0 [–0.6; +2.6]	+4.0 [+2.3; +5.8]	+1.6 [–0.3; +3.5]
91	+1.4 [–0.1; +2.9]	+1.1 [–1.9; +4.1]	–0.1 [–2.6; +2.6]	+1.5 [–0.7; +4.0]	+1.0 [–0.5; +2.6]	+0.9 [–0.7; +2.5]	+3.5 [+1.9; +5.1]	+1.4 [–0.4; +3.2]
92	+1.4 [–0.0; +2.9]	+0.8 [–2.1; +3.8]	–0.1 [–2.6; +2.5]	+1.0 [–1.1; +3.0]	+0.7 [–0.5; +2.1]	+0.6 [–0.7; +1.9]	+2.9 [+1.3; +4.5]	+1.2 [–0.5; +2.9]
93	+1.0 [–0.1; +2.4]	+0.4 [–1.8; +3.2]	–0.2 [–2.4; +2.1]	+0.4 [–0.9; +2.4]	+0.6 [–0.7; +1.8]	+0.3 [–0.9; +1.6]	+1.8 [+0.7; +3.2]	+1.0 [–0.4; +2.6]
94	+0.9 [–0.3; +2.0]	+0.2 [–2.0; +2.4]	–0.5 [–2.8; +1.7]	+0.7 [–0.7; +1.9]	+0.5 [–0.9; +1.9]	+0.1 [–0.6; +1.3]	+1.7 [+0.6; +2.7]	+0.7 [–0.6; +1.9]
95	+0.7 [–0.5; +1.9]	+0.1 [–2.0; +2.3]	–0.8 [–2.0; +1.5]	+1.0 [–0.5; +2.3]	+0.3 [–0.5; +1.1]	+0.1 [–0.6; +0.8]	+1.5 [+0.4; +2.6]	+0.6 [–0.8; +1.9]
96	+0.3 [–0.5; +1.0]	+0.1 [–1.1; +1.4]	–0.1 [–1.2; +0.9]	+1.0 [–0.2; +2.4]	+0.3 [–0.6; +1.1]	+0.1 [–0.6; +0.9]	+0.9 [+0.2; +1.7]	+0.3 [–0.7; +1.6]
97	+0.1 [–0.7; +0.8]	+0.3 [–1.0; +1.5]	+0.3 [–0.8; +1.3]	+0.8 [–0.2; +1.8]	+0.1 [–0.3; +0.9]	+0.1 [–0.2; +0.4]	+0.8 [+0.1; +1.5]	+0.1 [–0.9; +1.0]
98	–0.0 [–0.4; +0.2]	+0.2 [–0.4; +1.4]	+0.2 [–0.4; +1.1]	+0.8 [–0.2; +1.8]	+0.0 [–0.4; +0.5]	+0.1 [–0.2; +0.5]	+0.6 [+0.0; +1.2]	–0.1 [–0.5; +0.8]
99	–0.1 [–0.4; +0.3]	+0.2 [–0.4; +0.8]	+0.1 [–0.5; +0.8]	+0.4 [–0.0; +0.8]	–0.0 [–0.5; +0.4]	+0.2 [–0.2; +0.6]	+0.3 [+0.0; +0.6]	+0.0 [–0.4; +0.4]
100	–0.1 [–0.5; +0.3]	+0.3 [–0.4; +0.8]	+0.1 [–0.6; +0.7]	+0.5 [+0.0; +1.0]	–0.1 [–0.6; +0.3]	+0.2 [–0.2; +0.6]	+0.3 [–0.0; +0.7]	+0.1 [–0.4; +0.5]

Supplementary Table 7 Difference in quantile value between lockdown and expected at a fixed age at death, by region - Females (continued)

	**Quantile difference [95%Credibility Interval]**			
**Age**	*Normandie*	*Occitanie*	*Provence-Alpes-Côte d'Azur*	*Pays de la Loire*
40	-	-	-	-
41	-	-	+0.6 [–0.1; +1.5]	+0.6 [–0.2; +1.6]
42	+0.5 [–0.3; +1.7]	+0.5 [–0.3; +1.5]	+0.6 [–0.1; +1.5]	+0.6 [–0.2; +1.5]
43	+0.5 [–0.3; +1.6]	+0.4 [–0.3; +1.4]	+0.6 [–0.1; +1.4]	+0.5 [–0.2; +1.5]
44	+0.5 [–0.2; +1.5]	+0.4 [–0.3; +1.3]	+0.6 [–0.1; +1.4]	+0.5 [–0.2; +1.4]
45	+0.5 [–0.2; +1.5]	+0.4 [–0.3; +1.2]	+0.5 [–0.1; +1.3]	+0.5 [–0.1; +1.4]
46	+0.4 [–0.2; +1.4]	+0.3 [–0.3; +1.2]	+0.5 [–0.1; +1.3]	+0.5 [–0.1; +1.3]
47	+0.4 [–0.2; +1.3]	+0.3 [–0.3; +1.1]	+0.5 [–0.1; +1.2]	+0.5 [–0.1; +1.3]
48	+0.4 [–0.2; +1.2]	+0.2 [–0.3; +1.0]	+0.5 [–0.1; +1.2]	+0.5 [–0.1; +1.3]
49	+0.4 [–0.2; +1.2]	+0.2 [–0.3; +0.9]	+0.4 [–0.1; +1.1]	+0.5 [–0.1; +1.2]
50	+0.3 [–0.2; +1.1]	+0.2 [–0.3; +0.9]	+0.4 [–0.1; +1.0]	+0.5 [–0.1; +1.2]
51	+0.3 [–0.2; +1.0]	+0.1 [–0.3; +0.8]	+0.4 [–0.1; +1.0]	+0.5 [–0.1; +1.1]
52	+0.3 [–0.2; +0.9]	+0.1 [–0.3; +0.7]	+0.4 [–0.1; +0.9]	+0.4 [–0.1; +1.1]
53	+0.3 [–0.3; +0.9]	+0.1 [–0.4; +0.6]	+0.3 [–0.1; +0.9]	+0.4 [–0.1; +1.1]
54	+0.3 [–0.6; +0.9]	+0.0 [–0.8; +0.6]	+0.3 [–0.1; +0.8]	+0.4 [–0.0; +1.0]
55	+0.6 [–0.7; +1.2]	+0.0 [–1.0; +0.6]	+0.4 [–0.3; +0.9]	+0.7 [–0.1; +1.3]
56	+0.5 [–0.8; +1.5]	+0.0 [–0.9; +0.9]	+0.8 [–0.2; +1.2]	+1.0 [–0.1; +1.6]
57	+0.4 [–0.9; +1.7]	+0.1 [–0.8; +1.1]	+0.8 [–0.2; +1.6]	+1.2 [–0.1; +1.9]
58	+0.3 [–1.0; +1.6]	+0.1 [–0.8; +1.1]	+0.9 [–0.1; +1.9]	+1.2 [–0.1; +2.2]
59	+0.2 [–1.0; +1.5]	+0.2 [–0.7; +1.1]	+0.9 [–0.1; +1.9]	+1.2 [–0.0; +2.5]
60	+0.1 [–1.1; +1.4]	+0.2 [–0.7; +1.1]	+0.9 [–0.1; +1.9]	+1.2 [–0.0; +2.5]
61	–0.0 [–1.4; +1.2]	+0.2 [–0.6; +1.1]	+0.9 [–0.0; +1.9]	+1.1 [+0.0; +2.4]
62	–0.1 [–1.7; +1.1]	+0.3 [–0.5; +1.1]	+0.9 [+0.0; +1.9]	+1.1 [+0.0; +2.4]
63	–0.2 [–1.8; +1.2]	+0.3 [–0.7; +1.1]	+0.9 [+0.1; +1.9]	+1.1 [+0.1; +2.3]
64	–0.2 [–1.8; +1.4]	+0.5 [–0.8; +1.3]	+1.0 [+0.2; +2.0]	+1.3 [+0.1; +2.5]
65	–0.2 [–1.7; +1.4]	+0.5 [–0.8; +1.7]	+1.5 [+0.1; +2.4]	+1.7 [+0.0; +2.8]
66	–0.1 [–1.7; +1.4]	+0.6 [–0.7; +1.9]	+1.7 [+0.1; +2.8]	+1.8 [–0.1; +3.1]
67	–0.1 [–1.7; +1.4]	+0.6 [–0.7; +1.9]	+1.6 [+0.1; +3.2]	+1.7 [–0.1; +3.4]
68	–0.1 [–1.7; +1.5]	+0.6 [–0.7; +1.9]	+1.5 [+0.0; +3.1]	+1.6 [–0.2; +3.4]
69	–0.1 [–1.7; +1.5]	+0.6 [–0.6; +1.9]	+1.5 [–0.0; +3.0]	+1.5 [–0.2; +3.3]
70	–0.1 [–1.7; +1.5]	+0.6 [–0.6; +1.9]	+1.4 [–0.0; +2.9]	+1.4 [–0.3; +3.2]

Supplementary Table 7 Difference in quantile value between lockdown and expected at a fixed age at death, by region - Females (continued)

	**Quantile difference [95%Credibility Interval]**			
**Age**	*Normandie*	*Occitanie*	*Provence-Alpes-Côte d'Azur*	*Pays de la Loire*
71	−0.1 [−1.6; +1.5]	+0.6 [−0.6; +1.9]	+1.3 [−0.1; +2.8]	+1.3 [−0.4; +3.1]
72	−0.1 [−2.0; +1.5]	+0.7 [−0.6; +1.9]	+1.3 [−0.1; +2.8]	+1.2 [−0.4; +3.0]
73	−0.1 [−2.4; +1.5]	+0.7 [−0.5; +1.9]	+1.2 [−0.1; +2.7]	+1.1 [−0.5; +2.9]
74	−0.1 [−2.3; +2.1]	+0.7 [−0.6; +1.9]	+1.1 [−0.3; +2.6]	+1.1 [−0.6; +2.8]
75	−0.1 [−2.2; +2.5]	+1.1 [−0.8; +2.3]	+1.7 [−0.4; +3.1]	+1.4 [−0.8; +3.1]
76	−0.1 [−2.0; +2.3]	+1.2 [−0.8; +3.0]	+1.6 [−0.5; +3.7]	+1.6 [−0.7; +3.7]
77	−0.1 [−1.9; +2.1]	+1.1 [−0.7; +3.3]	+1.4 [−0.5; +3.7]	+1.6 [−0.6; +4.3]
78	−0.1 [−1.8; +2.0]	+1.1 [−0.7; +3.1]	+1.2 [−0.6; +3.4]	+1.5 [−0.5; +4.0]
79	−0.1 [−2.2; +1.8]	+1.0 [−0.6; +2.8]	+1.0 [−0.7; +3.0]	+1.5 [−0.4; +3.8]
80	−0.2 [−2.7; +2.1]	+0.9 [−0.5; +2.6]	+0.8 [−1.1; +2.7]	+1.4 [−0.2; +3.5]
81	−0.1 [−2.5; +2.6]	+1.3 [−1.0; +2.9]	+1.1 [−1.3; +3.3]	+1.9 [−0.5; +3.7]
82	−0.1 [−2.2; +2.4]	+1.2 [−1.0; +3.9]	+0.8 [−1.4; +3.4]	+2.1 [−0.7; +4.7]
83	−0.1 [−2.5; +2.2]	+0.9 [−1.1; +3.3]	+0.5 [−1.5; +2.8]	+1.5 [−1.0; +4.5]
84	−0.2 [−2.7; +2.7]	+0.7 [−1.4; +2.7]	+0.3 [−1.9; +2.4]	+1.0 [−1.6; +3.7]
85	−0.3 [−2.6; +2.3]	+0.6 [−1.7; +3.0]	+0.2 [−1.8; +2.5]	+0.8 [−1.9; +3.9]
86	−0.4 [−2.8; +2.0]	+0.3 [−1.8; +2.5]	+0.2 [−1.6; +2.3]	+0.3 [−2.0; +3.1]
87	−0.2 [−2.5; +2.2]	+0.0 [−2.1; +2.2]	−0.0 [−2.2; +2.4]	−0.1 [−2.5; +2.5]
88	−0.0 [−2.3; +2.2]	−0.1 [−2.1; +2.0]	−0.5 [−2.5; +1.8]	−0.3 [−2.6; +2.2]
89	−0.1 [−2.4; +2.3]	−0.1 [−2.1; +1.9]	−0.8 [−2.7; +1.2]	−0.4 [−2.8; +2.0]
90	−0.2 [−2.3; +2.0]	+0.1 [−1.7; +2.0]	−0.9 [−2.7; +1.1]	−0.5 [−2.7; +1.8]
91	−0.1 [−2.1; +1.8]	+0.3 [−1.3; +2.1]	−0.8 [−2.5; +0.9]	−0.7 [−2.9; +1.5]
92	+0.1 [−1.8; +2.0]	+0.5 [−1.0; +2.1]	−0.7 [−2.3; +1.0]	−1.1 [−3.3; +1.0]
93	+0.0 [−1.4; +1.6]	+0.5 [−0.7; +2.0]	−0.5 [−1.7; +0.9]	−1.0 [−2.4; +0.4]
94	−0.1 [−1.5; +1.4]	+0.5 [−0.8; +1.7]	−0.4 [−1.7; +0.8]	−1.0 [−2.4; +0.4]
95	−0.2 [−1.2; +1.2]	+0.5 [−0.9; +1.9]	−0.4 [−1.7; +0.8]	−1.0 [−2.3; +0.4]
96	−0.1 [−0.9; +0.8]	+0.3 [−0.6; +1.4]	−0.2 [−1.0; +0.5]	−0.5 [−1.3; +0.3]
97	+0.0 [−0.8; +0.9]	+0.1 [−0.7; +1.0]	−0.1 [−0.9; +0.7]	−0.4 [−1.2; +0.5]
98	+0.0 [−0.5; +0.4]	+0.0 [−0.3; +0.6]	+0.1 [−0.4; +0.9]	−0.1 [−0.5; +0.6]
99	−0.0 [−0.6; +0.5]	+0.0 [−0.4; +0.4]	+0.1 [−0.3; +0.5]	+0.0 [−0.5; +0.5]
100	−0.1 [−0.8; +0.5]	+0.0 [−0.5; +0.4]	+0.2 [−0.3; +0.6]	+0.2 [−0.4; +0.7]

Supplementary Figure 1 Expected age at death without lockdown and observed age with lockdown for each quantile in Auvergne-Rhône-Alpes

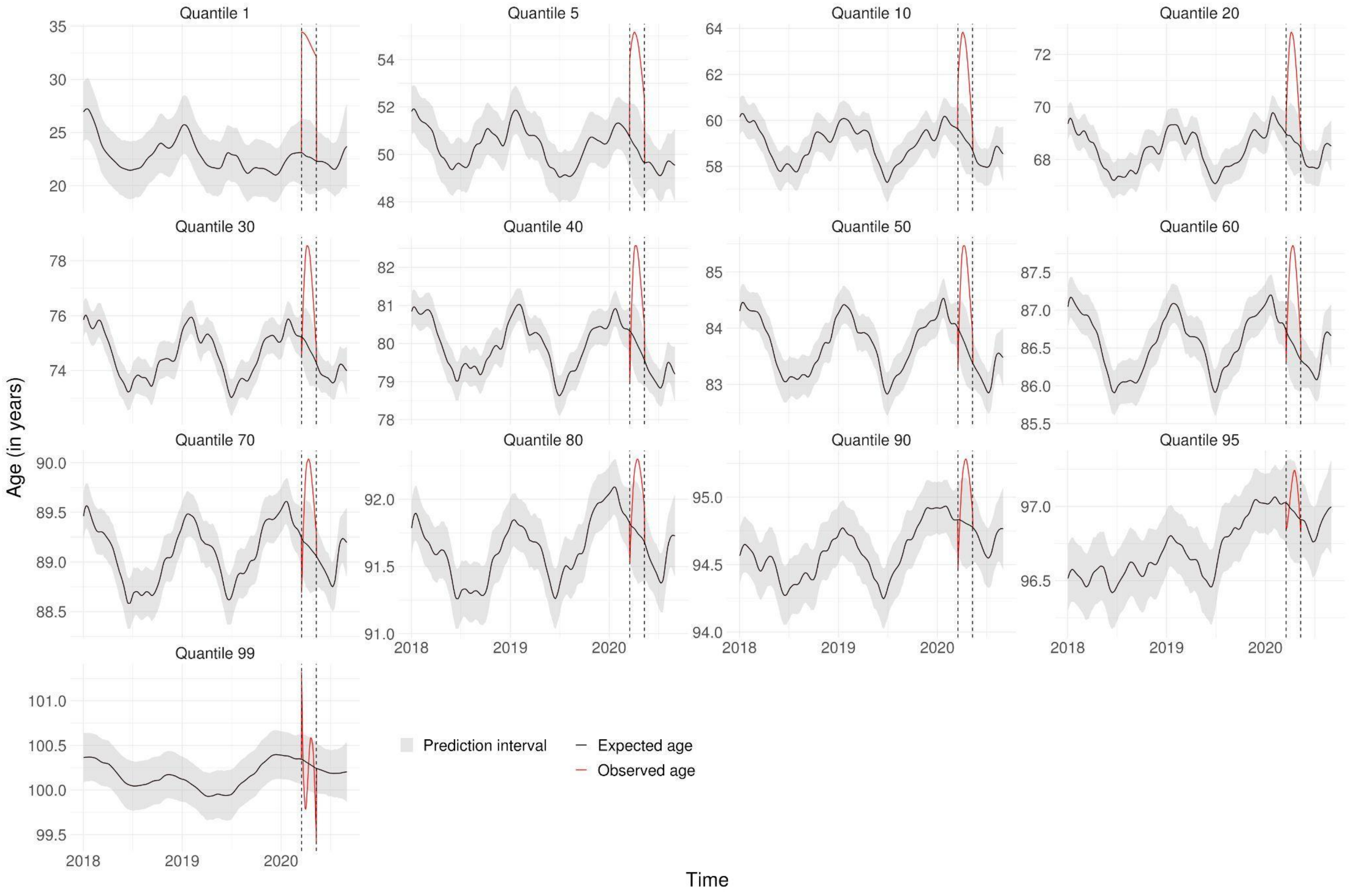

Supplementary Figure 2 Expected age at death without lockdown and observed age with lockdown for each quantile in Centre-Val de Loire

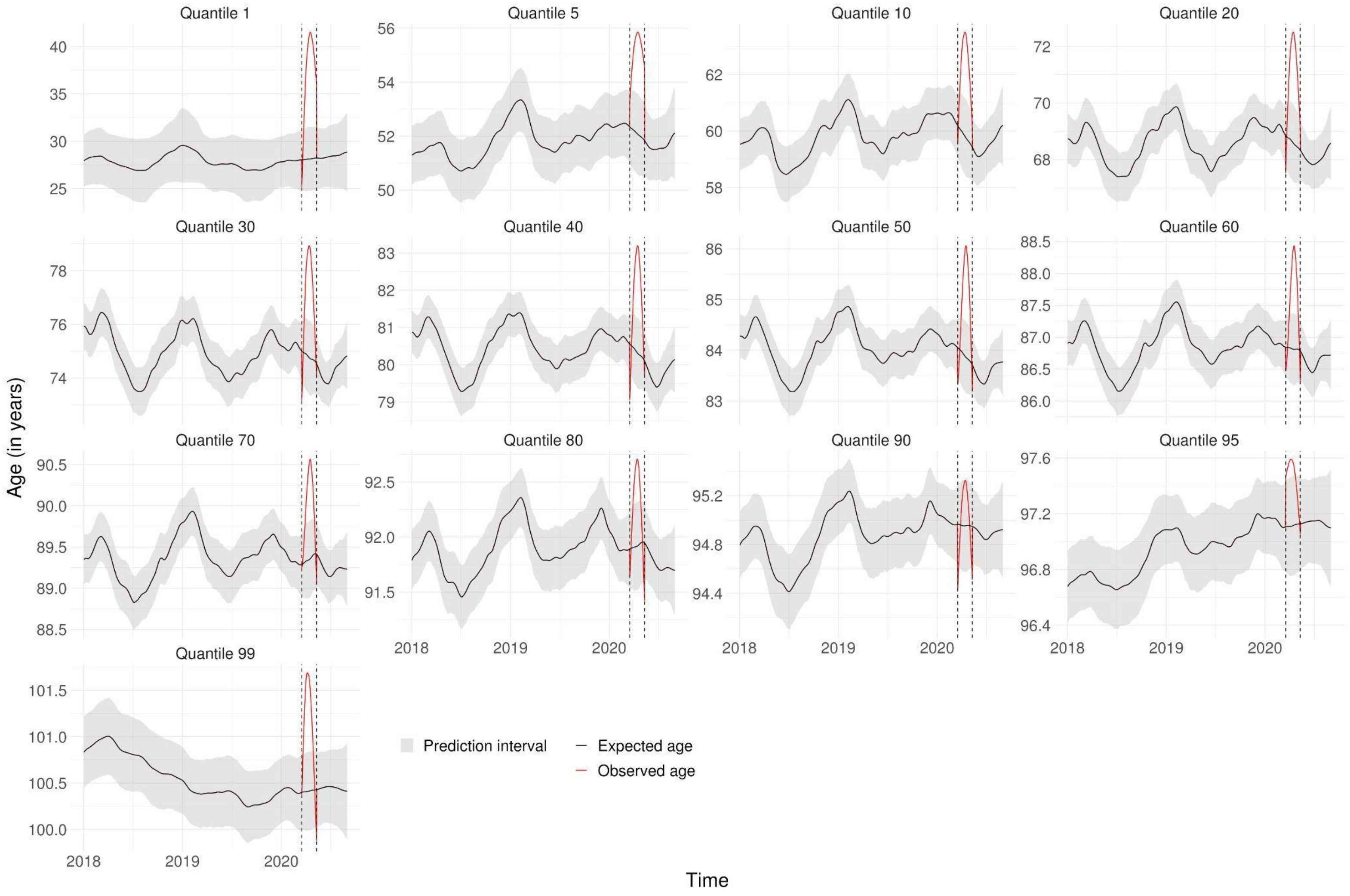

Supplementary Figure 3 Expected age at death without lockdown and observed age with lockdown for each quantile in Grand-Est

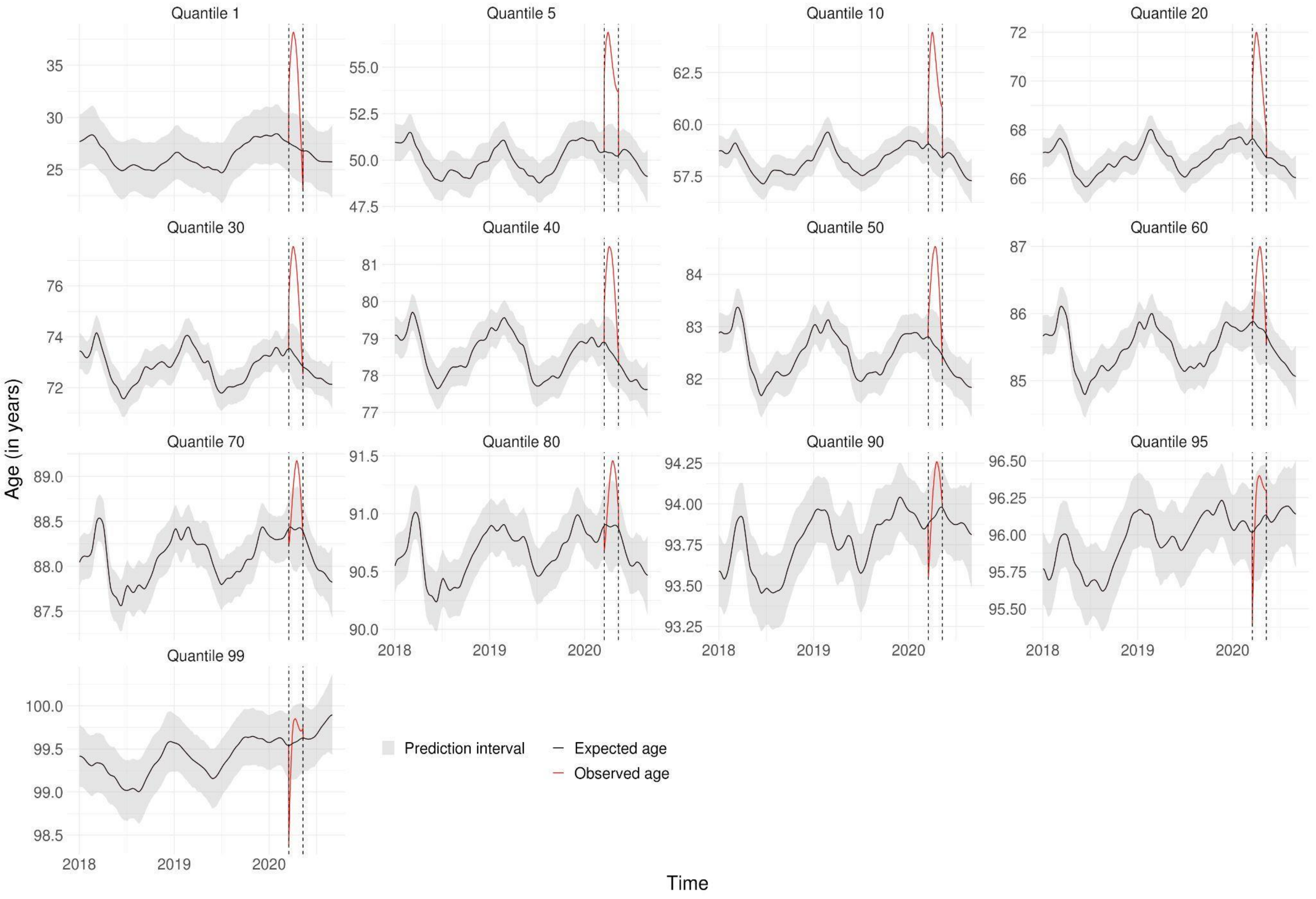

Supplementary Figure 4 Expected age at death without lockdown and observed age with lockdown for each quantile in Hauts-de-France

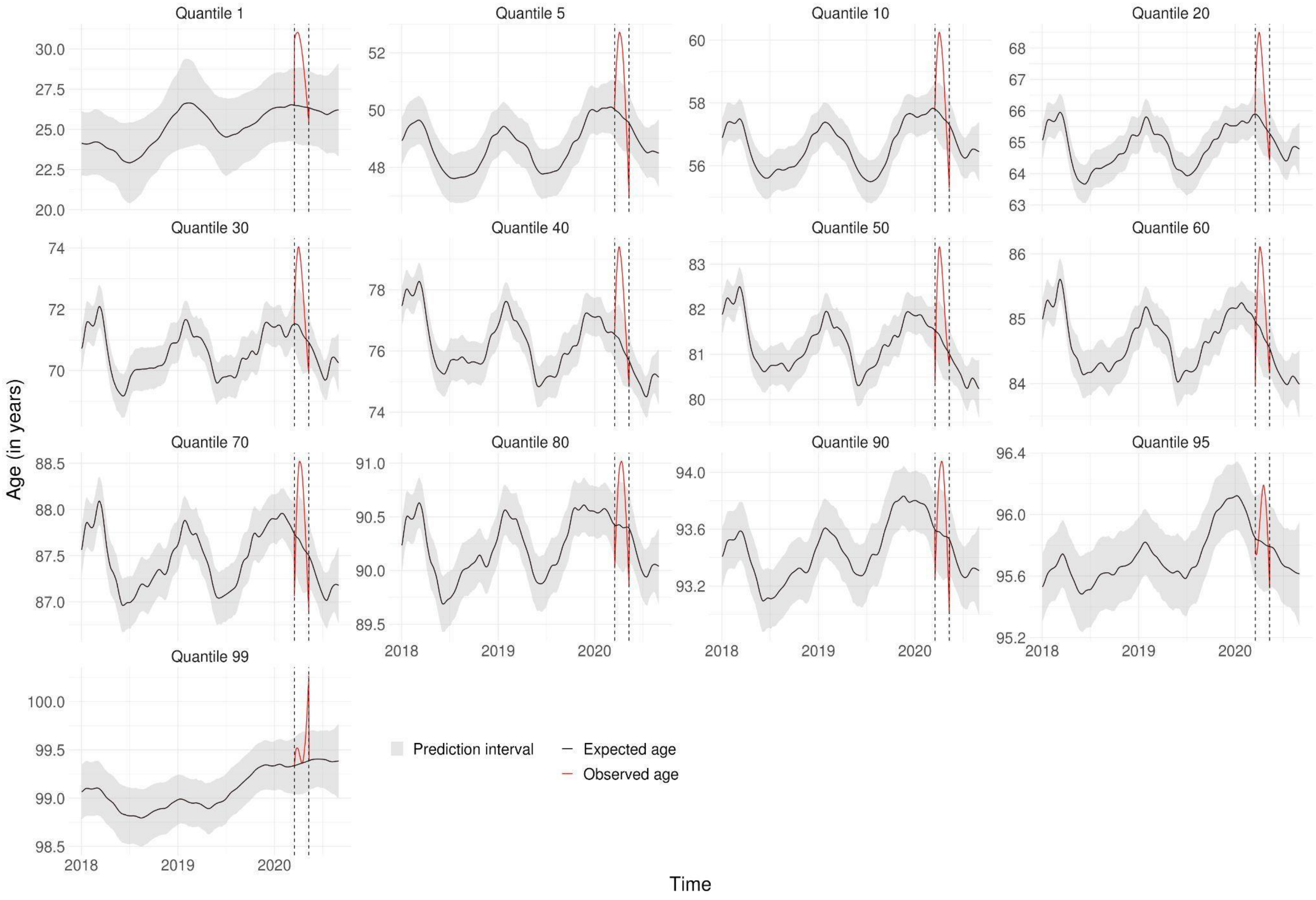

Supplementary Figure 5 Expected age at death without lockdown and observed age with lockdown for each quantile in Normandie

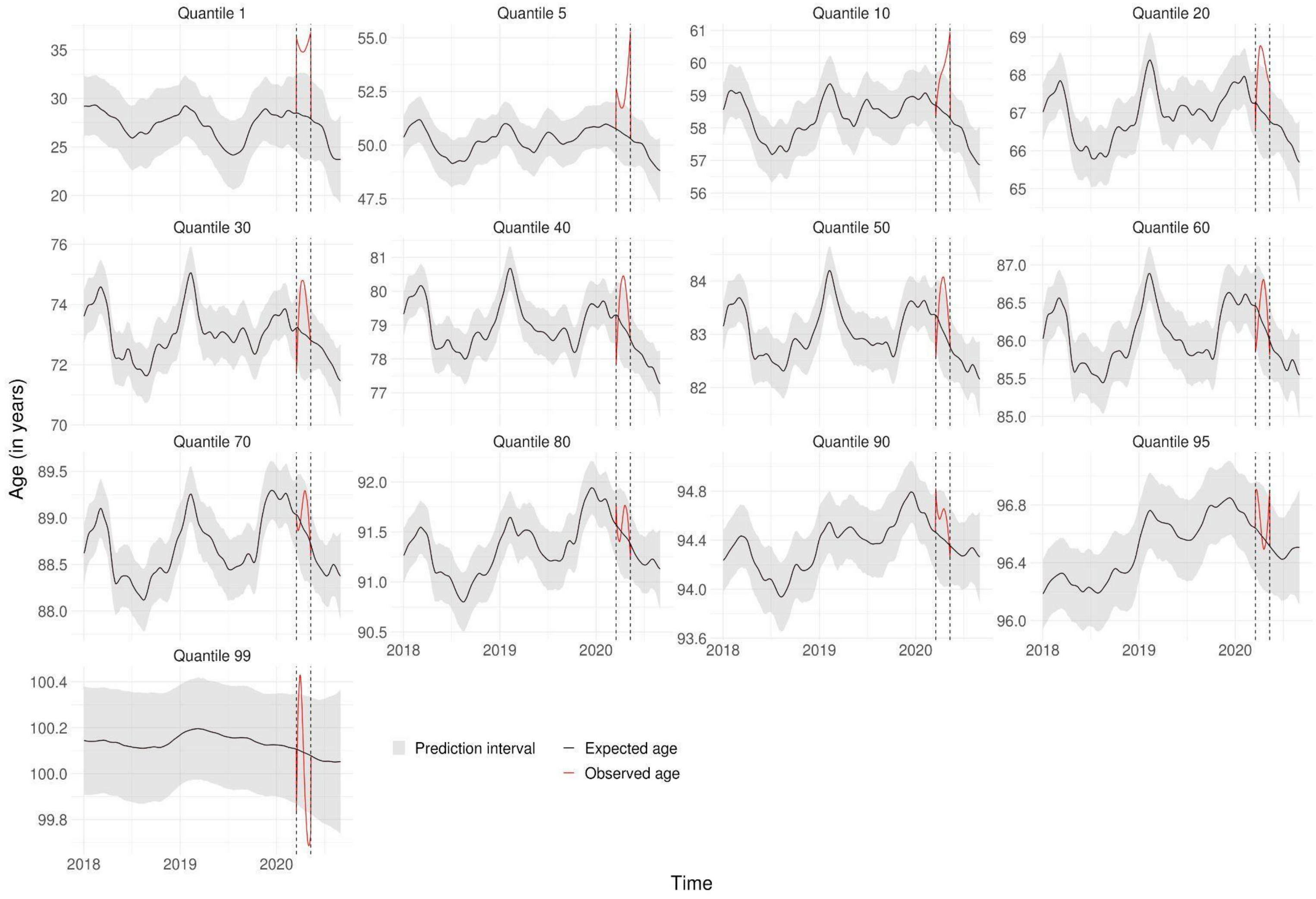

Supplementary Figure 6 Expected age at death without lockdown and observed age with lockdown for each quantile in Nouvelle Aquitaine

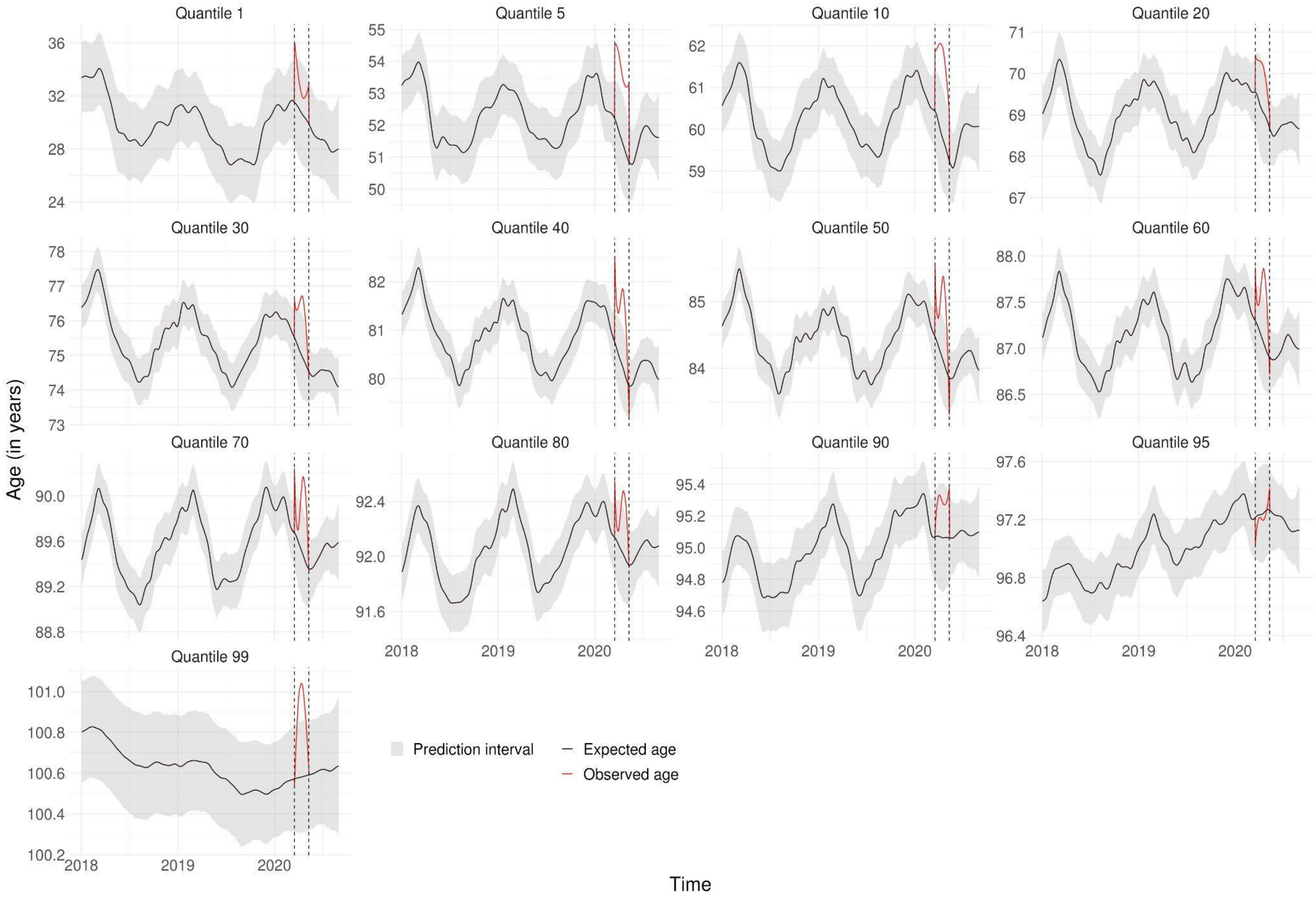

Supplementary Figure 7 Expected age at death without lockdown and observed age with lockdown for each quantile in Occitanie

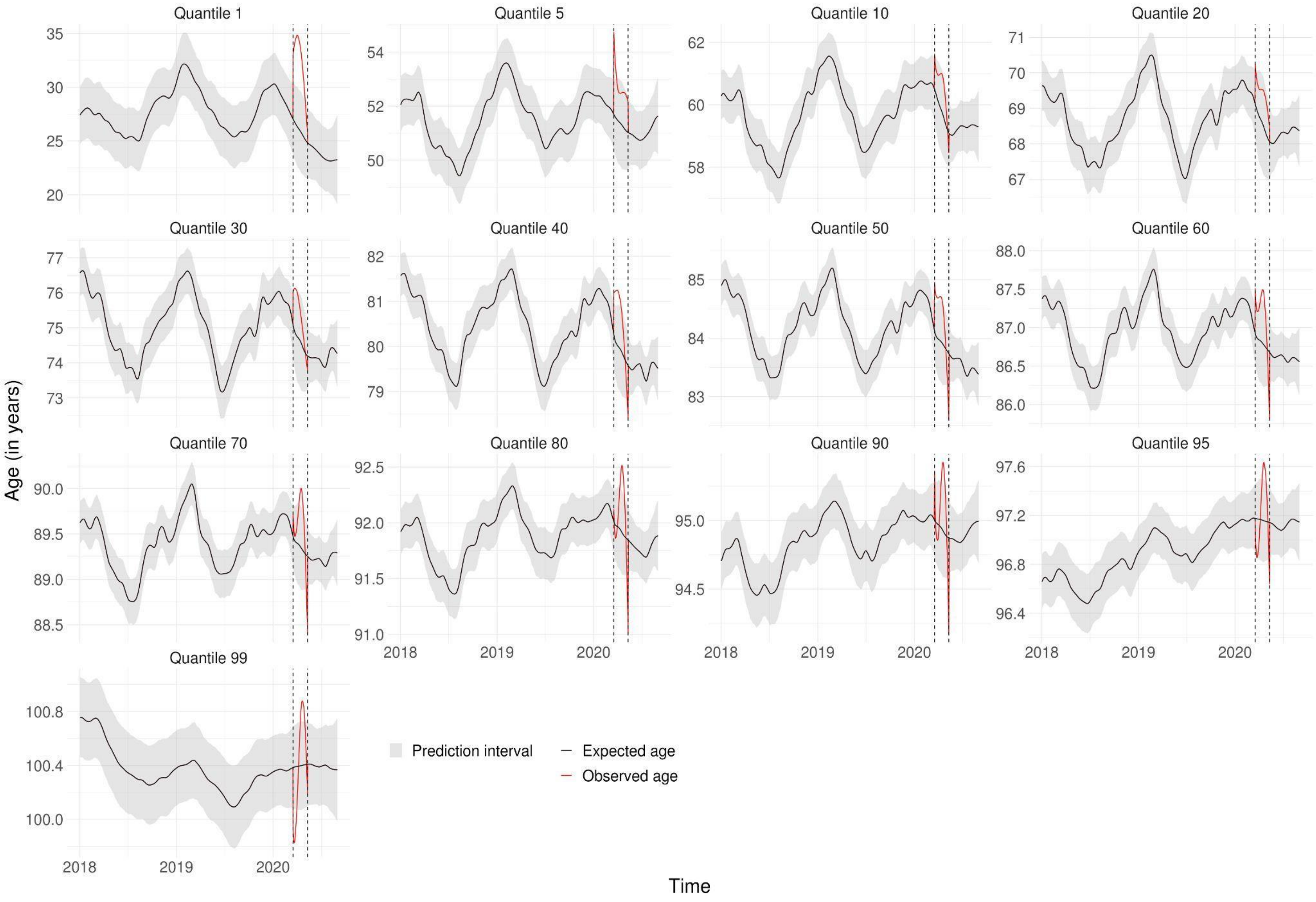

Supplementary Figure 8 Expected age at death without lockdown and observed age with lockdown for each quantile in Pays de la Loire

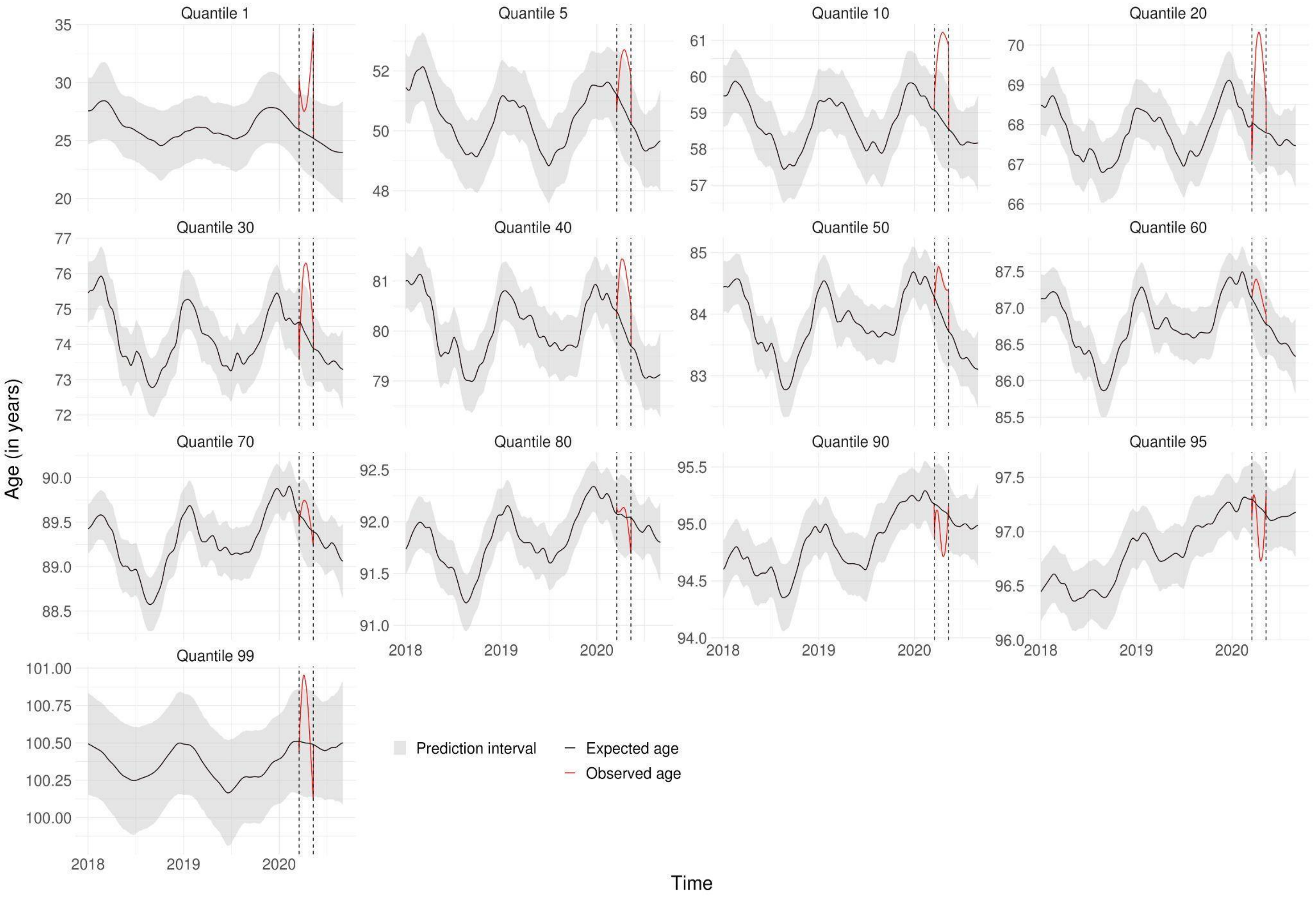

Supplementary Figure 9 Expected age at death without lockdown and observed age with lockdown for each quantile in Provence-Alpes-Côte d'Azur

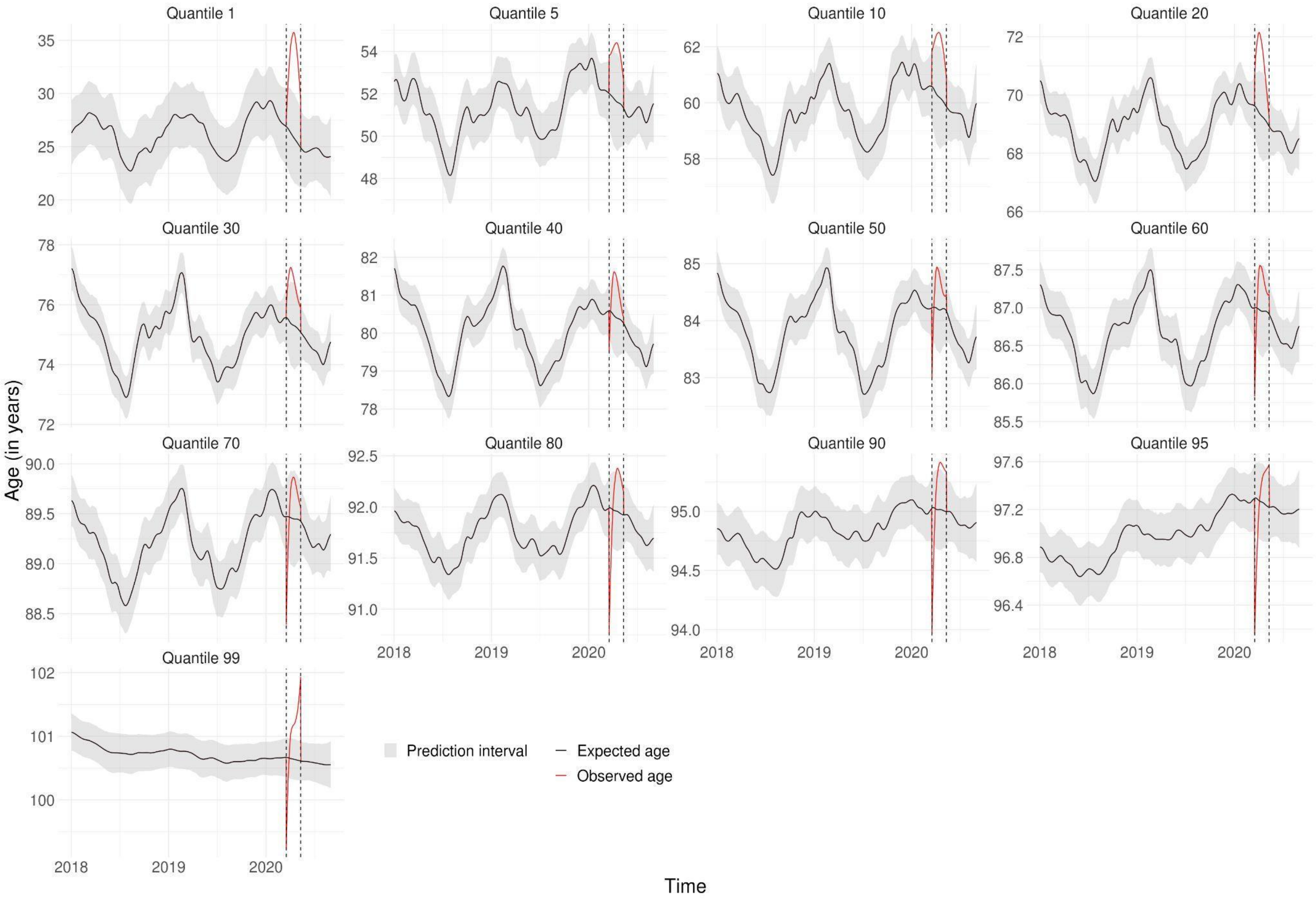

Supplementary Figure 10 Evolution of the number of deaths according to age at death and sex during the lockdown in 2020 and the corresponding period in 2017-2019 in Auvergne-Rhône-Alpes

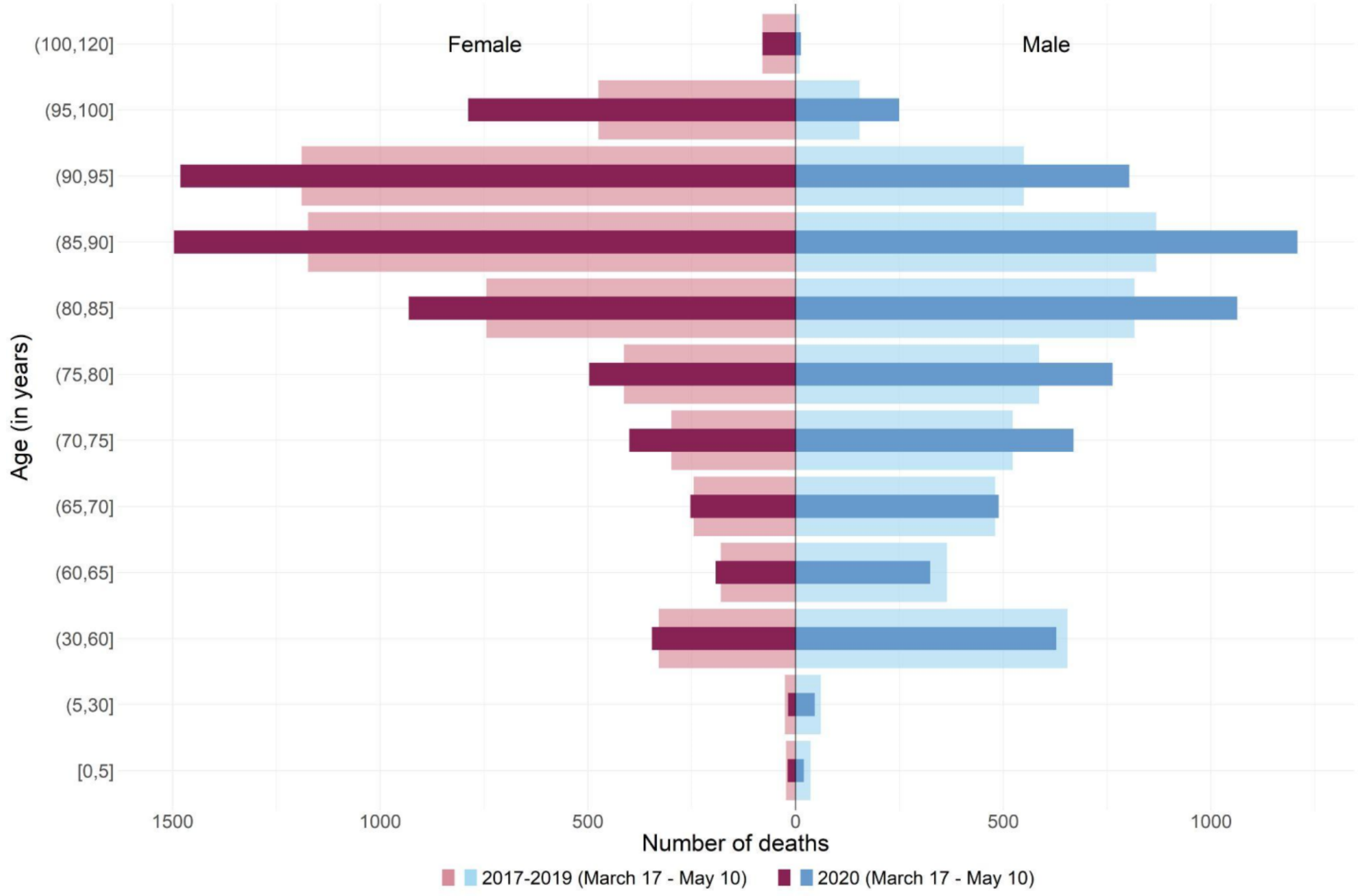

Dark red and blue stand for 2020 and light red and blue stand for 2017-2019

Supplementary Figure 11 Evolution of the number of deaths according to age at death and sex during the lockdown in 2020 and the corresponding period in 2017-2019 in Centre-Val de Loire

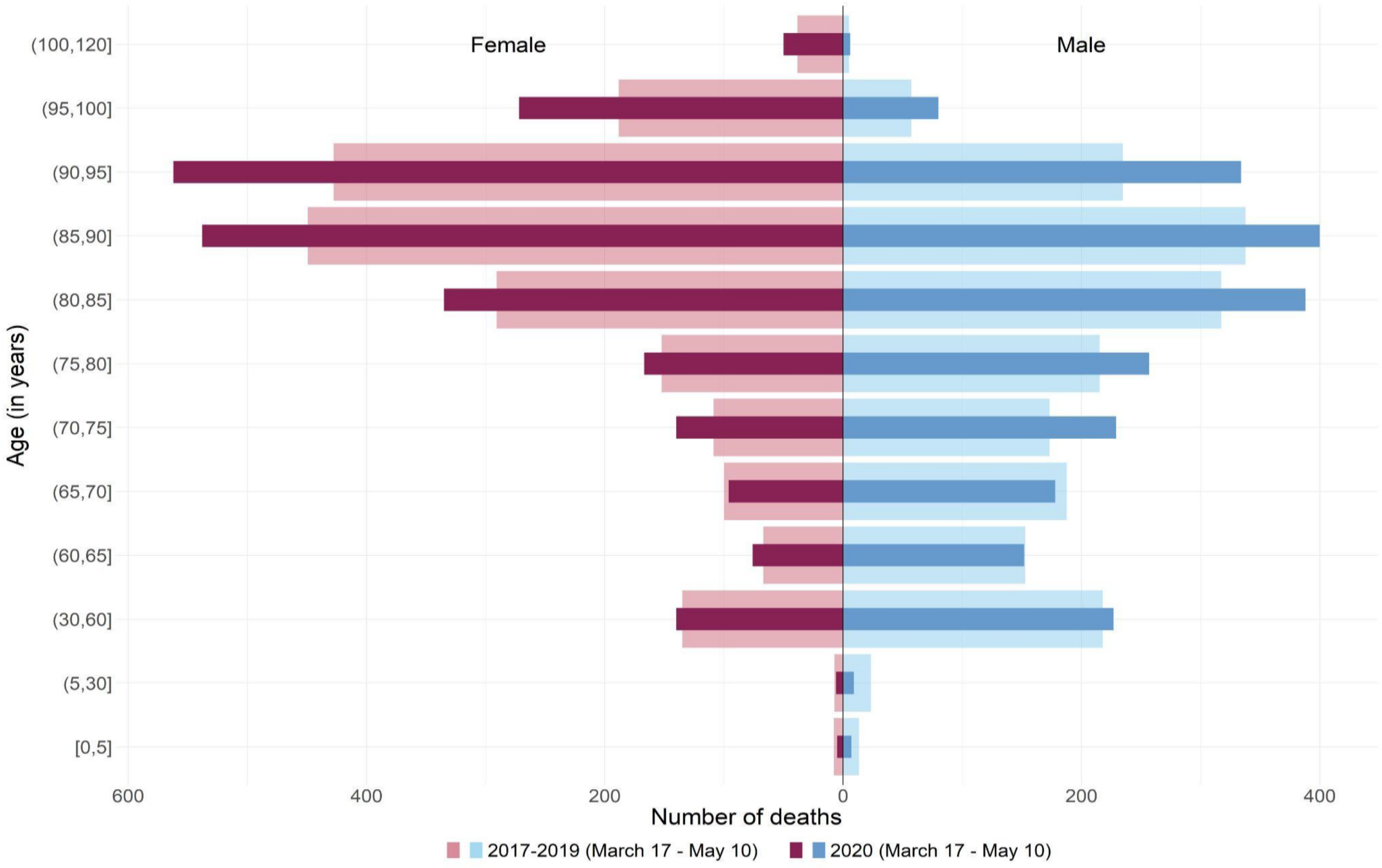

Dark red and blue stand for 2020 and light red and blue stand for 2017-2019

Supplementary Figure 12 Evolution of the number of deaths according to age at death and sex during the lockdown in 2020 and the corresponding period in 2017-2019 in Grand-Est

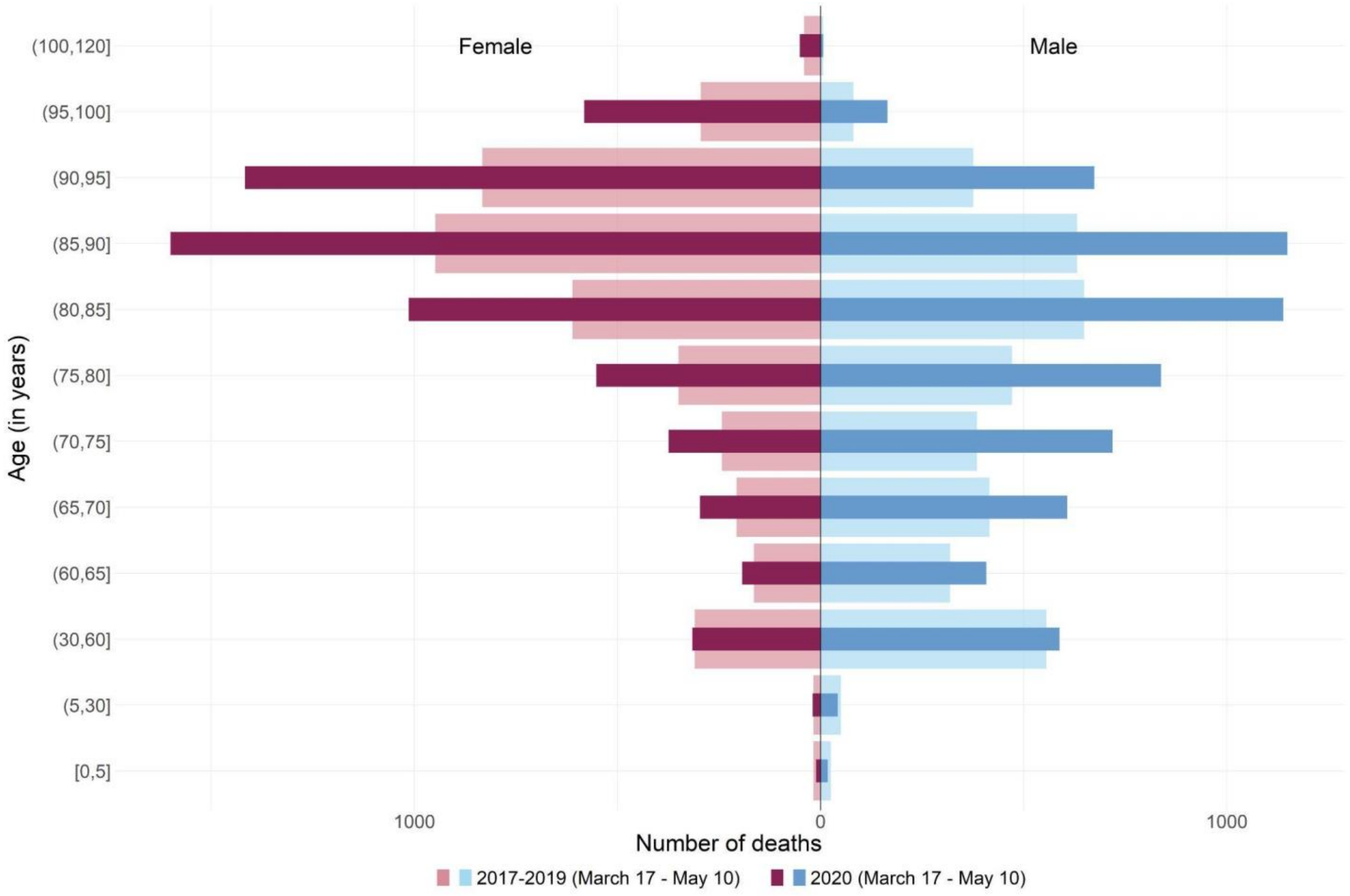

Dark red and blue stand for 2020 and light red and blue stand for 2017-2019

Supplementary Figure 13 Evolution of the number of deaths according to age at death and sex during the lockdown in 2020 and the corresponding period in 2017-2019 in Hauts-de-France

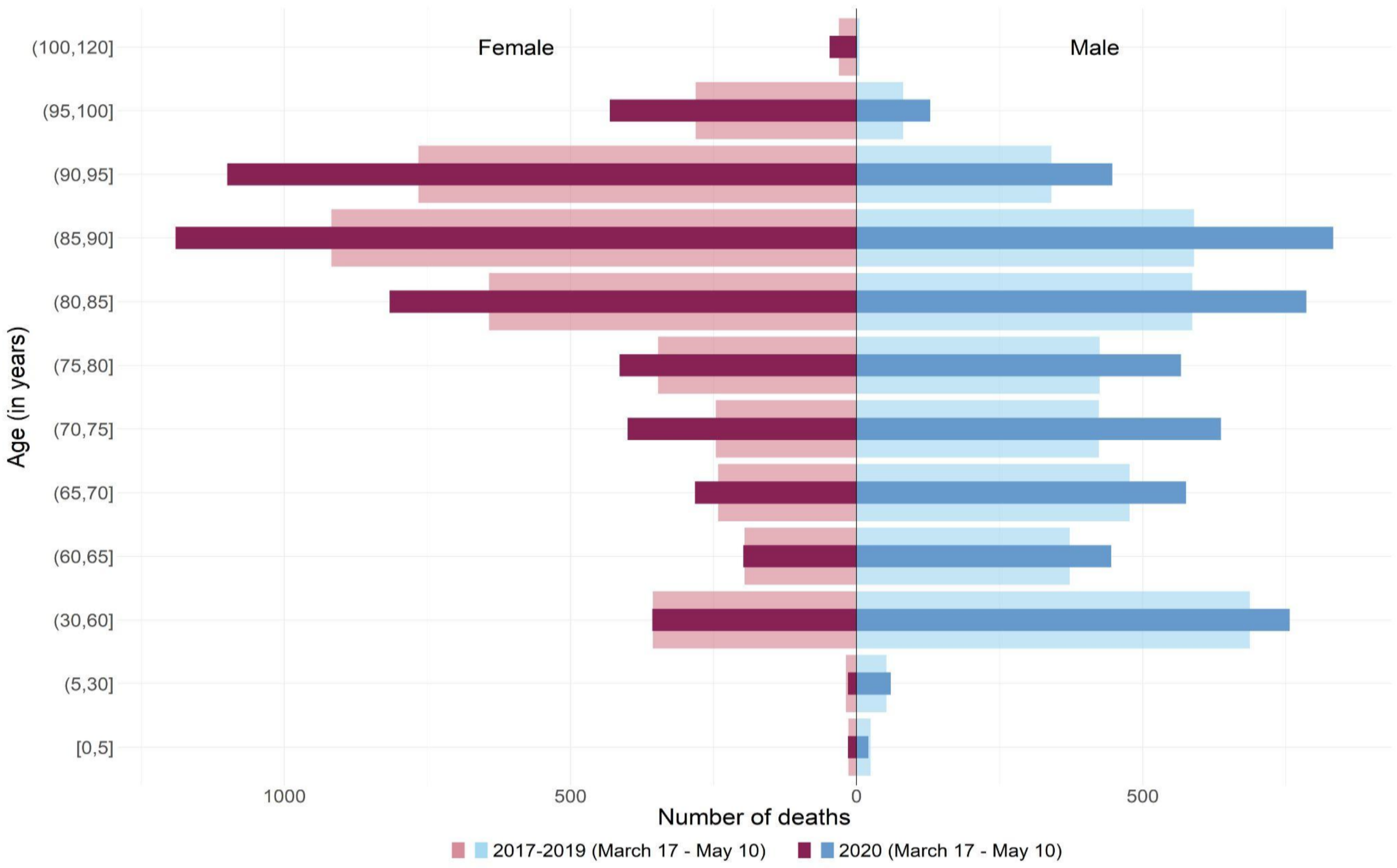

Dark red and blue stand for 2020 and light red and blue stand for 2017-2019

Supplementary Figure 14 Evolution of the number of deaths according to age at death and sex during the lockdown in 2020 and the corresponding period in 2017-2019 in Normandie

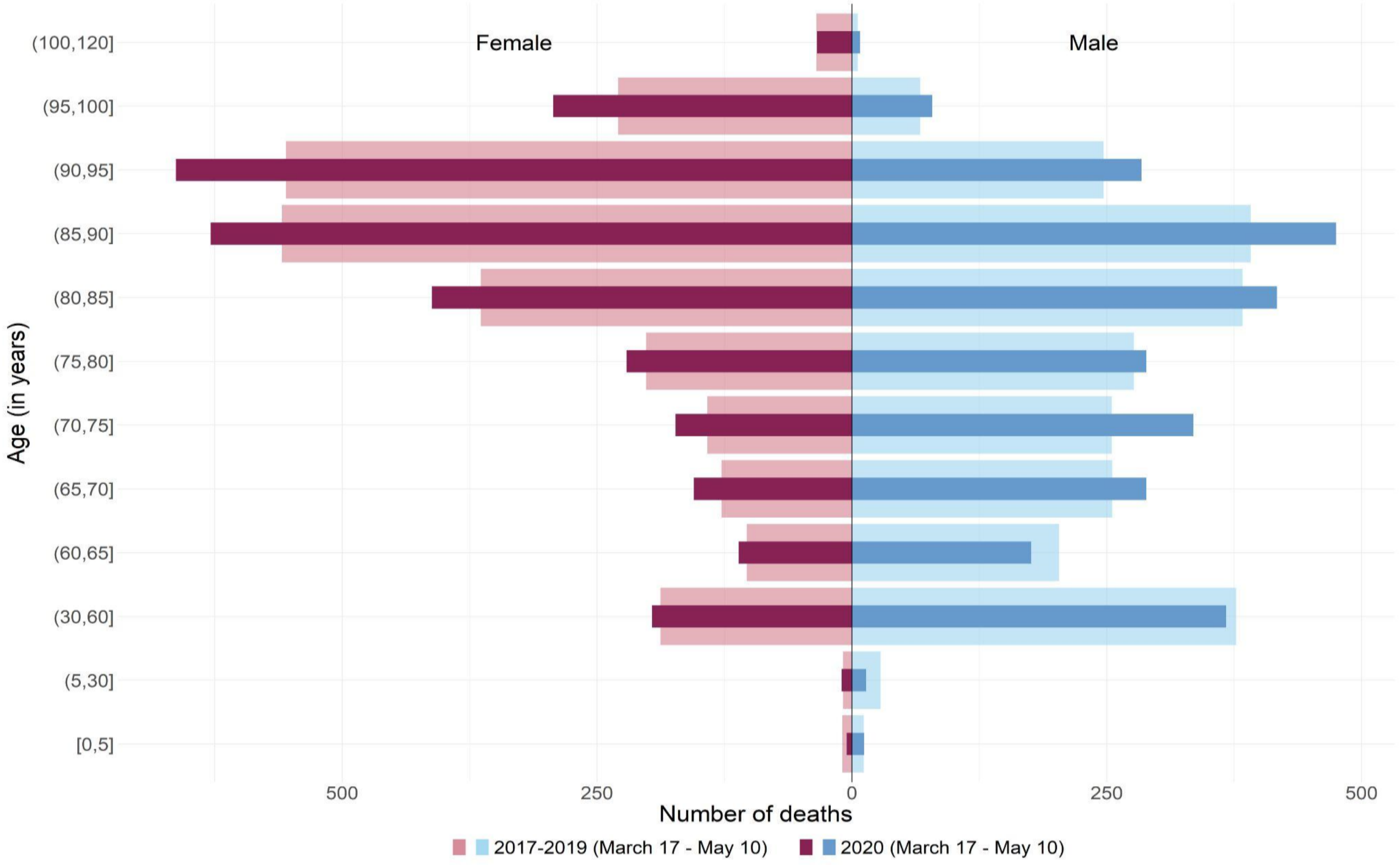

Dark red and blue stand for 2020 and light red and blue stand for 2017-2019

Supplementary Figure 15 Evolution of the number of deaths according to age at death and sex during the lockdown in 2020 and the corresponding period in 2017-2019 in Nouvelle Aquitaine

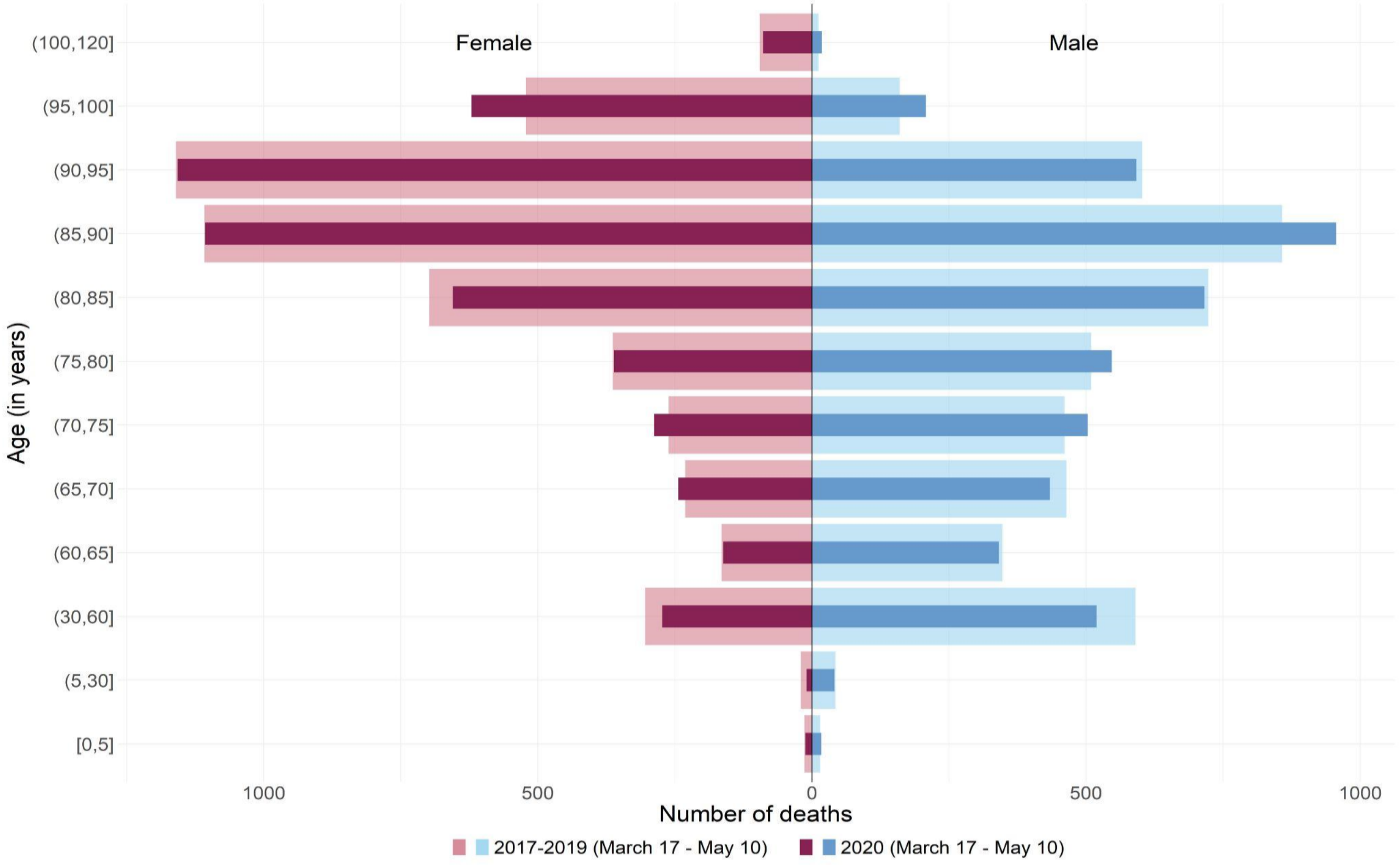

Dark red and blue stand for 2020 and light red and blue stand for 2017-2019

Supplementary Figure 16 Evolution of the number of deaths according to age at death and sex during the lockdown in 2020 and the corresponding period in 2017-2019 in Occitanie

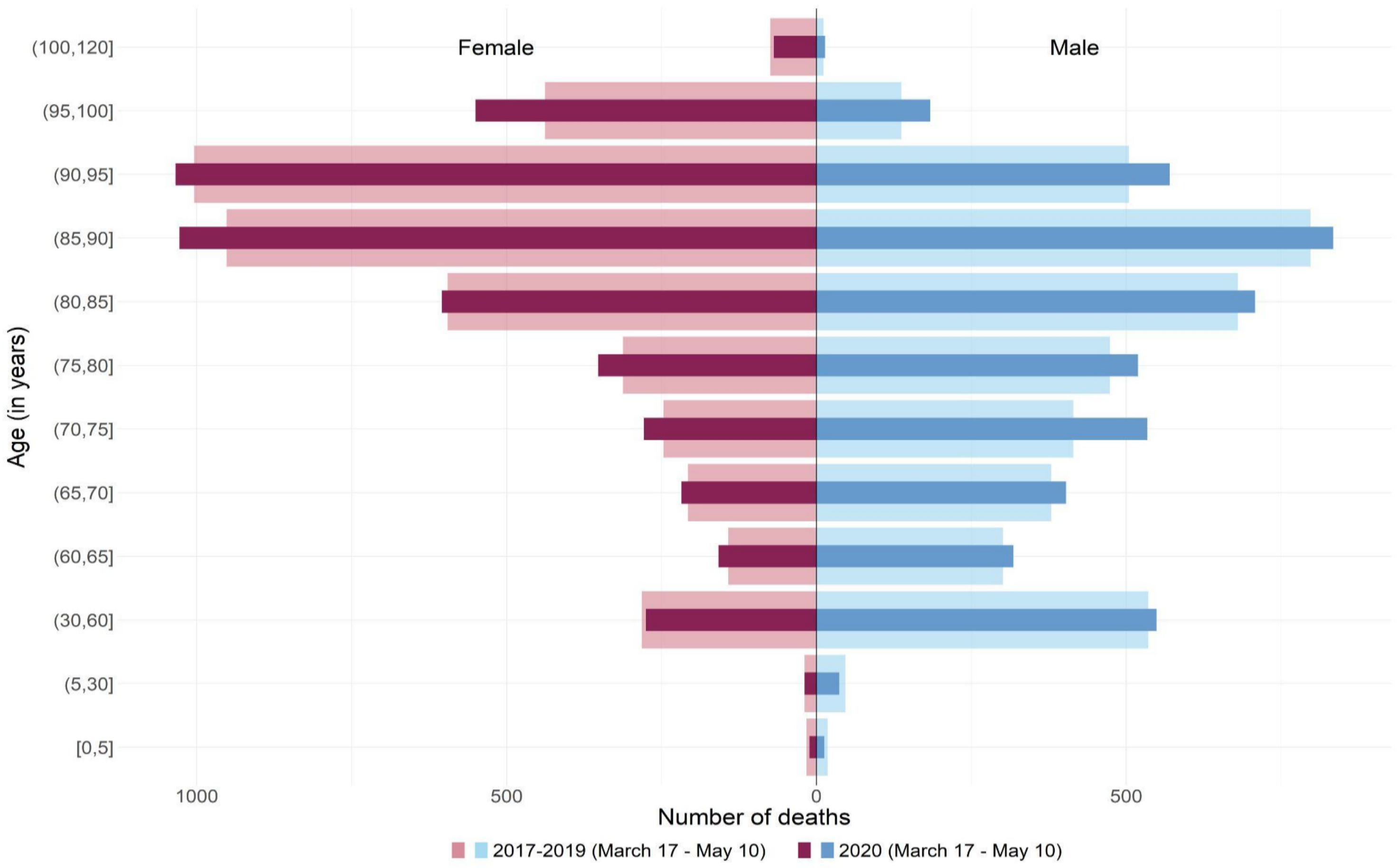

Dark red and blue stand for 2020 and light red and blue stand for 2017-2019

Supplementary Figure 17 Evolution of the number of deaths according to age at death and sex during the lockdown in 2020 and the corresponding period in 2017-2019 in Pays de la Loire

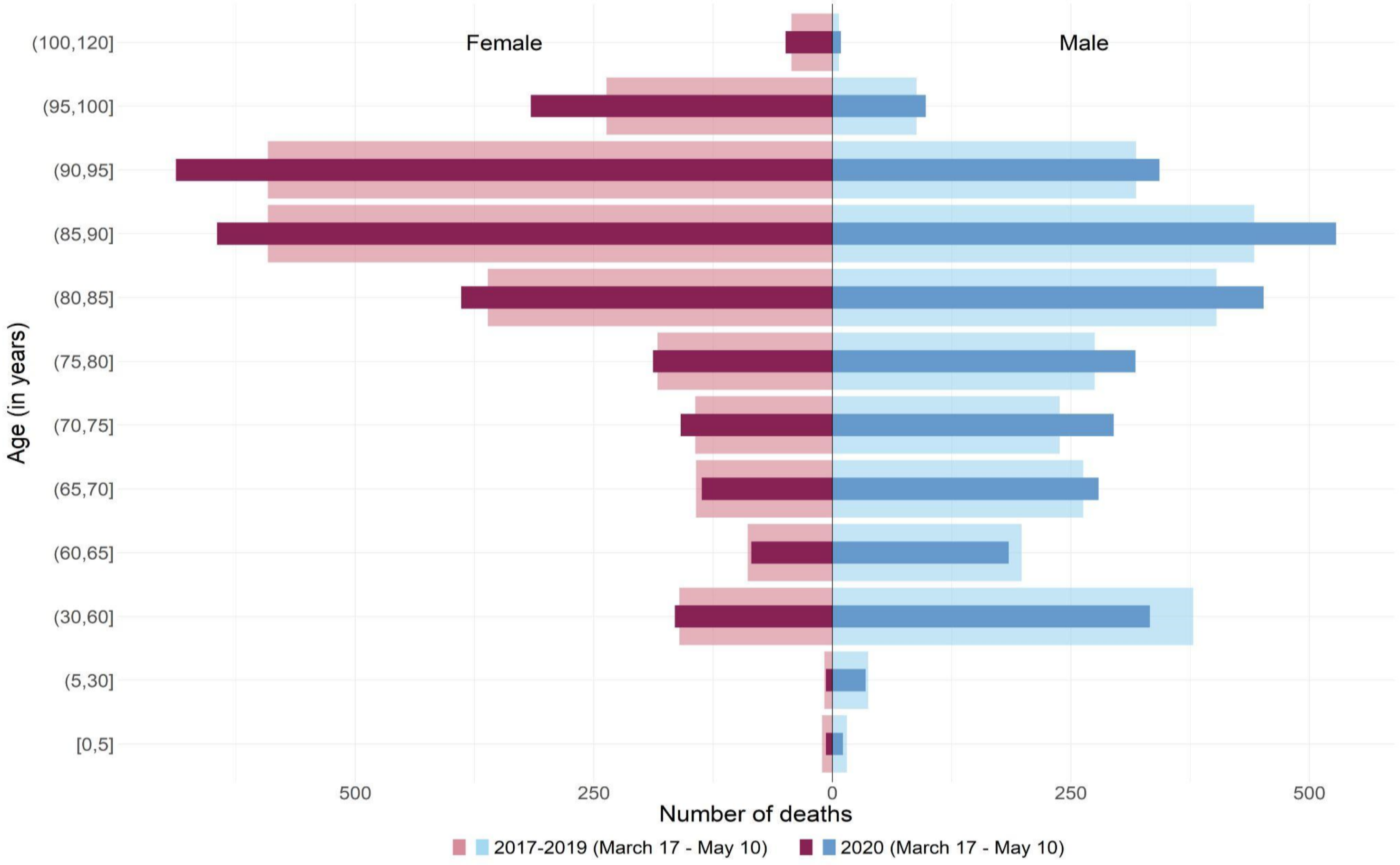

Dark red and blue stand for 2020 and light red and blue stand for 2017-2019

Supplementary Figure 18 Evolution of the number of deaths according to age at death and sex during the lockdown in 2020 and the corresponding period in 2017-2019 in Provence-Alpes-Côte d'Azur

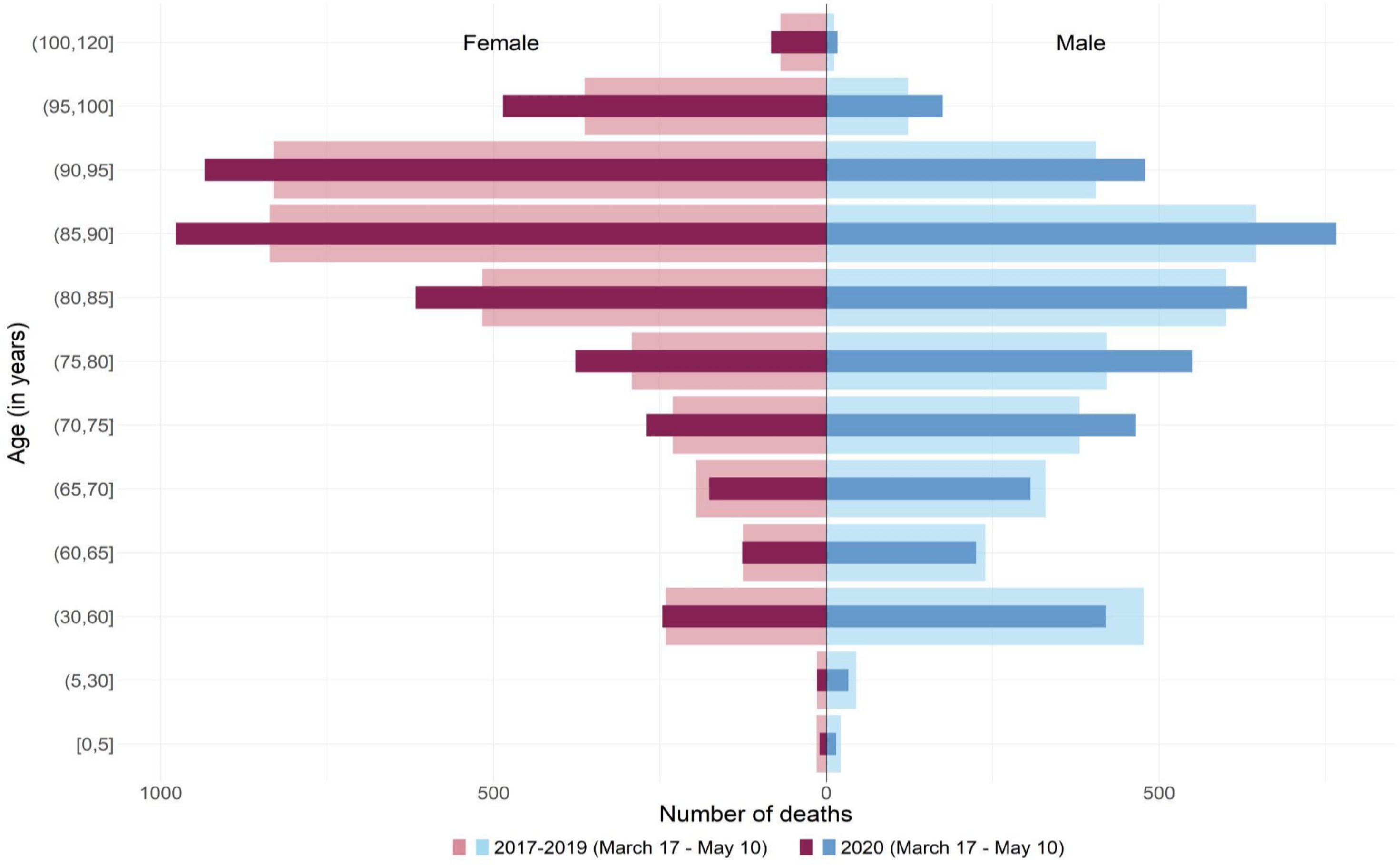

Dark red and blue stand for 2020 and light red and blue stand for 2017-2019
